# JCGA: the Japanese version of the Cancer Genome Atlas and its contribution to the interpretation of gene alterations detected in clinical cancer genome sequencing

**DOI:** 10.1038/s41439-021-00170-w

**Published:** 2021-09-30

**Authors:** Masakuni Serizawa, Maki Mizuguchi, Kenichi Urakami, Takeshi Nagashima, Keiichi Ohshima, Keiichi Hatakeyama, Sumiko Ohnami, Shumpei Ohnami, Koji Maruyama, Tadashi Ashizawa, Akira Iizuka, Yasue Horiuchi, Akane Naruoka, Hirotsugu Kenmotsu, Yasuto Akiyama, Ken Yamaguchi

**Affiliations:** 1 Drug Discovery and Development Division, Shizuoka Cancer Center Research Institute, Shizuoka, Japan; 2 Division of Thoracic Oncology, Shizuoka Cancer Center Hospital, Shizuoka, Japan; 3 Shizuoka Cancer Center, Shizuoka, Japan; 4 Cancer Diagnostics Research Division, Shizuoka Cancer Center Research Institute, Shizuoka, Japan; 5 SRL, Tokyo, Japan; 6 Medical Genetics Division, Shizuoka Cancer Center Research Institute, Shizuoka, Japan; 7 Experimental Animal Facility, Shizuoka Cancer Center Research Institute, Shizuoka, Japan; 8 Immunotherapy Division, Shizuoka Cancer Center Research Institute, Shizuoka, Japan; 9 Division of Genetic Medicine Promotion, Shizuoka Cancer Center Hospital, Shizuoka, Japan

**Keywords:** Cancer genomics, Cancer genomics

## Abstract

With the emergence of next-generation sequencing (NGS)-based cancer gene panel tests in routine oncological practice in Japan, an easily interpretable cancer genome database of Japanese patients in which mutational profiles are unaffected by racial differences is needed to improve the interpretation of the detected gene alterations. Considering this, we constructed the first Japanese cancer genome database, called the Japanese version of the Cancer Genome Atlas (JCGA), which includes multiple tumor types. The database includes whole-exome sequencing data from 4907 surgically resected primary tumor samples obtained from 4753 Japanese patients with cancer and graphically provides genome information on 460 cancer-associated genes, including the 336 genes that are included in two NGS-based cancer gene panel tests approved by the Pharmaceuticals and Medical Devices Agency. Moreover, most of the contents of this database are written in Japanese; this not only helps physicians explain the results of NGS-based cancer gene panel tests but also enables patients and their families to obtain further information regarding the detected gene alterations.

In oncological practice, next-generation sequencing (NGS)-based cancer gene panel tests provide information regarding gene alterations that aids in the selection of appropriate treatment regimens or promising clinical trials for patients and improves the understanding of the biological drivers of tumor growth and progression^1,2^. NGS-based cancer gene panel tests were launched as part of routine oncological practice in Japan in June 2019, and their costs are reimbursed by the national insurance system^3^. Between June 2019 and June 2021, a total of 18,239 patients underwent NGS-based cancer gene panel tests^4^. The members of molecular tumor boards, which are also known as expert panels, have interpreted the detected gene alterations to discuss the available genetically informed treatment options. However, these panels have relied on databases such as the OncoKB^5^, DoCM^6^, CIViC^7^, and several commercially available databases^8,9^, which have been constructed mainly based on cancer genome information collected from Caucasian patients. Moreover, the distribution of histological types and mutational frequency reportedly varies among ethnic groups^10–14^. Therefore, a cancer genome database of multiple tumor types that is based on information collected from Japanese patients is needed.

Here, we describe a Japanese cancer genome database of multiple tumor types, called the Japanese version of the Cancer Genome Atlas (JCGA), that graphically provides whole-exome sequencing data from 4907 fresh-frozen surgically resected primary tumor tissues. These samples were obtained from 4753 Japanese patients with cancer who were enrolled in Project HOPE (High-tech Omics-based Patient Evaluation), a prospective molecular profiling study for multiple tumor types using multiomics technology centered on NGS that was launched at the Shizuoka Cancer Center in January 2014 (Shizuoka Cancer Center institutional review board authorization number #25‐33)^15^. This cohort consisted of 134 tumor types classified based on the criteria of Oncotree^16^ and/or The Cancer Genome Atlas^17^ (Supplementary Table S1). The first version of the JCGA provided information on 460 cancer-associated genes, including 336 genes that are involved in two NGS-based cancer gene panel tests approved by the Pharmaceuticals and Medical Devices Agency (PMDA) (Supplementary Table S2). These genes were classified into 11 functional categories and 27 signaling pathways (Supplementary Table S3). Most of the contents of the JCGA are written in Japanese; this helps physicians explain the results of NGS-based cancer gene panel tests and also enables patients and their families to obtain further information regarding the detected gene alterations. Somatic gene alterations in the JCGA, which were identified according to the procedure described by Nagashima et al.^14^, are available from the National Bioscience Database Center Human Database (Research ID, hum0127) as VCF or TSV format files^18^.

Users can search the JCGA by entering the official gene symbol, conventional gene symbol, or official gene ID in the search window of the homepage (Fig. 1). Moreover, a gene grid function (a clickable gene list) provided at the bottom of the homepage can facilitate further browsing (Fig. 1). The resultant display page of each gene provides information regarding eight content items (Fig. 1), which are divided into two sections: (A) the gene information section (content items 1–4), which provides biological background for each gene, and (B) the whole-exome sequencing data section (content items 5–8), which graphically provides the whole-exome sequencing data for the 4907 primary tumor samples in Project HOPE. The content details are as follows: (1) basic information, such as links to relevant bioinformatic resources and gene maps showing the chromosomal location, exon–intron organization, and coding region; (2) the gene summary, which explains the biological function of the gene and its associations with carcinogenesis and tumor progression; (3) a list of PMDA-approved drugs targeting the selected gene, which is updated quarterly depending on the approval status; (4) a pathway map, which shows the association between cancer signaling pathways and the selected gene; (5) graphs that indicate the frequency of somatic alterations of this gene in 30 principal tumor types, listed in Supplementary Table S1; (6) the distribution of tumor mutational burden (TMB) in 30 principal tumor types (Supplementary Table S1) sorted in ascending order of the median value of TMB in each tumor type, as described in Supplementary Table S4; (7) plots of the distribution of mutations in the linear protein sequence, i.e., the so-called lollipop plots; and (8) a list of driver somatic mutations. A detailed explanation of the homepage and the contents on the gene display page are provided in the Supplementary Material.

**Fig. 1:**
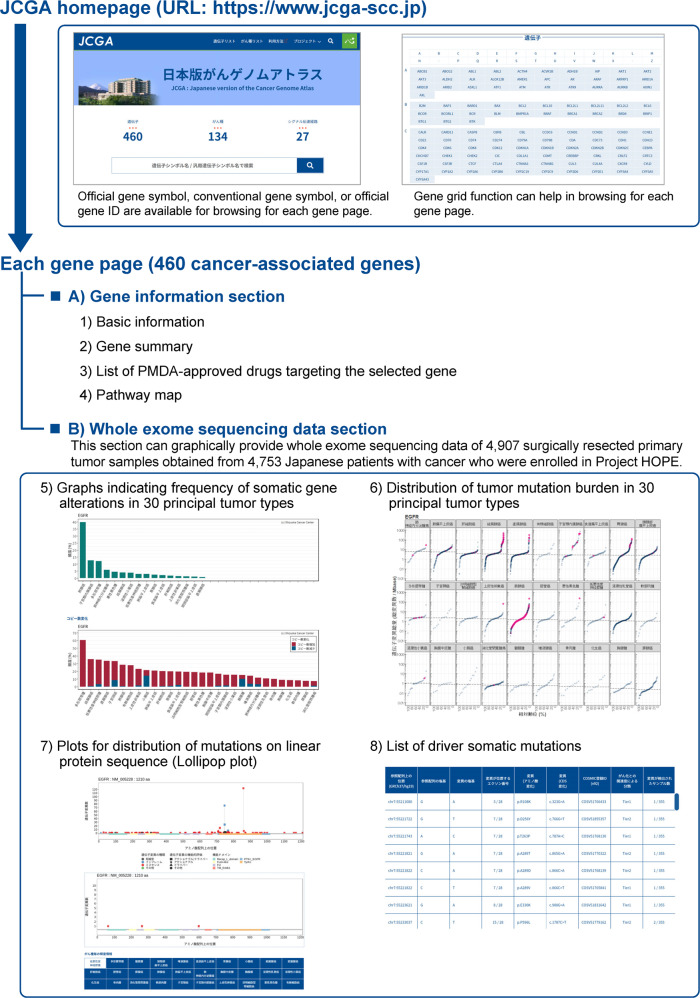
Overview of the Japanese version of the Cancer Genome Atlas. Users can search by entering the official gene symbol, conventional gene symbol, or official gene ID (Entrez ID) in the search window or gene grid function. The JCGA provides information on 460 cancer-associated genes (Supplementary Table S2). The resultant display page of each gene provides data about the eight content items, which are classified into two sections. The gene information section (content items 1–4) provides the biological background, and the whole-exome sequencing data section (content items 5–8) graphically provides whole-exome sequencing data from Project HOPE. A detailed explanation of the homepage and the contents on the gene display page are provided in the Supplementary Material.

The JCGA has been used by the expert panel at our institute for the interpretation of gene alterations detected in NGS-based cancer gene panel tests and for the preparation of reports that explain putative biological causes that drive tumor growth and progression. The graphs showing the frequency of somatic gene alterations (Fig. 1 (5)) can contribute to the estimation of the primary tumor type or its site based on the evaluation of the commonality of tumor types harboring mutated genes in the patient with an unknown primary tumor. The TMB distribution (Fig. 1 (6)) can be used to assess the relative ranking of the TMB values in patients who have undergone NGS-based gene panel testing from each Japanese patient population comprising subjects with 30 principal tumor types, listed in Supplementary Table S1. This is useful in predicting the therapeutic effect of immune checkpoint inhibitors^19^. The TMB distribution also contributes to the simultaneous evaluation of i) tumor types for which alterations in the selected genes are frequently observed in patients and ii) the effects of these alterations on the TMB. Lollipop plots (Fig. 1 (7)) are effective for identifying putative functional mutations located in functional domains and mutational hotspots, which are recurrently mutated positions that are frequently observed in oncogenes. The “comparison of lollipop plots” function can be used on the JCGA to compare the distribution of the detected mutations in the linear protein sequence between all tumor types (upper plot) and the selected tumor types (lower plot) and contributes to the identification of tumor types that frequently harbor mutations in particular hotspots or functional domains.

This version of the JCGA is limited in its ability to provide Japanese patient cancer genome information to cancer researchers worldwide, as it is written in Japanese. Therefore, an English version of the JCGA is currently under construction and is scheduled for release in 2022. Project HOPE is a single-institute study; thus, genome information and clinical records can be accessed and correlated for each patient. Therefore, future versions of the JCGA can provide information regarding the prognosis and treatment outcomes of patients associated with their gene alterations.

## Software availability

The JCGA is available through the following URL: https://www.jcga-scc.jp.

## Supplementary information

Supplementary Material. A detailed explanation of the homepage and the contents on the gene display page

Supplementary Table S1. Tumor types in JCGA (134) classified according to Oncotree and/or The Cancer Genome Atlas (TCGA) criteria.

Supplementary Table S2. Information in JCGA for 460 cancer-associated genes

Supplementary Table S3. List of functional categories and signaling pathways

Supplementary Table S4. Median tumor mutation burden (TMB) in each principal tumor type
