# Supplementary material for: JCGA: the Japanese version of the Cancer Genome Atlas and its contribution to the interpretation of gene alterations detected in clinical cancer genome sequencing
Source: Hum Genome Var. 2021 Sep 30;8:38. doi: 10.1038/s41439-021-00170-w (PMC8481308; doi:10.1038/s41439-021-00170-w)
Supplement: Supplementary file 1 — Supplementary Material. A detailed explanation of the homepage and the contents on the gene display page [file 41439_2021_170_MOESM1_ESM.pdf]

Supplementary Material

JCGA: The Japanese version of the Cancer Genome Atlas and its contribution to the interpretation of gene alterations detected in clinical cancer genome sequencing

Serizawa *et al.*

Table of contents

	Page
1. Homepage of the Japanese version of the Cancer Genome Atlas	1
2. The resulting view of each gene page from a keyword search	
1) Basic information	3
2) Gene summary	6
3) List of Pharmaceuticals and Medical Devices Agency (PMDA)-approved drugs targeting the selected gene	7
4) Pathway map	8
5) Graphs indicating the frequency of somatic gene alterations in 30 principal tumor types	9
6) Distribution of tumor mutation burden in 30 principal tumor types	10
7) Plots for distribution of mutations on linear protein sequence	11
8) List of driver somatic mutations	12

1. Homepage of the Japanese version of the Cancer Genome Atlas

- Users can search by entering either the official gene symbol, trivial gene symbol, or official gene ID (Entrez ID) in the search window or gene grid function. Links to the lists of 460 genes (Table S2), 134 tumor types (Table S1), and functional categories and signaling pathways (Table S3) are provided.

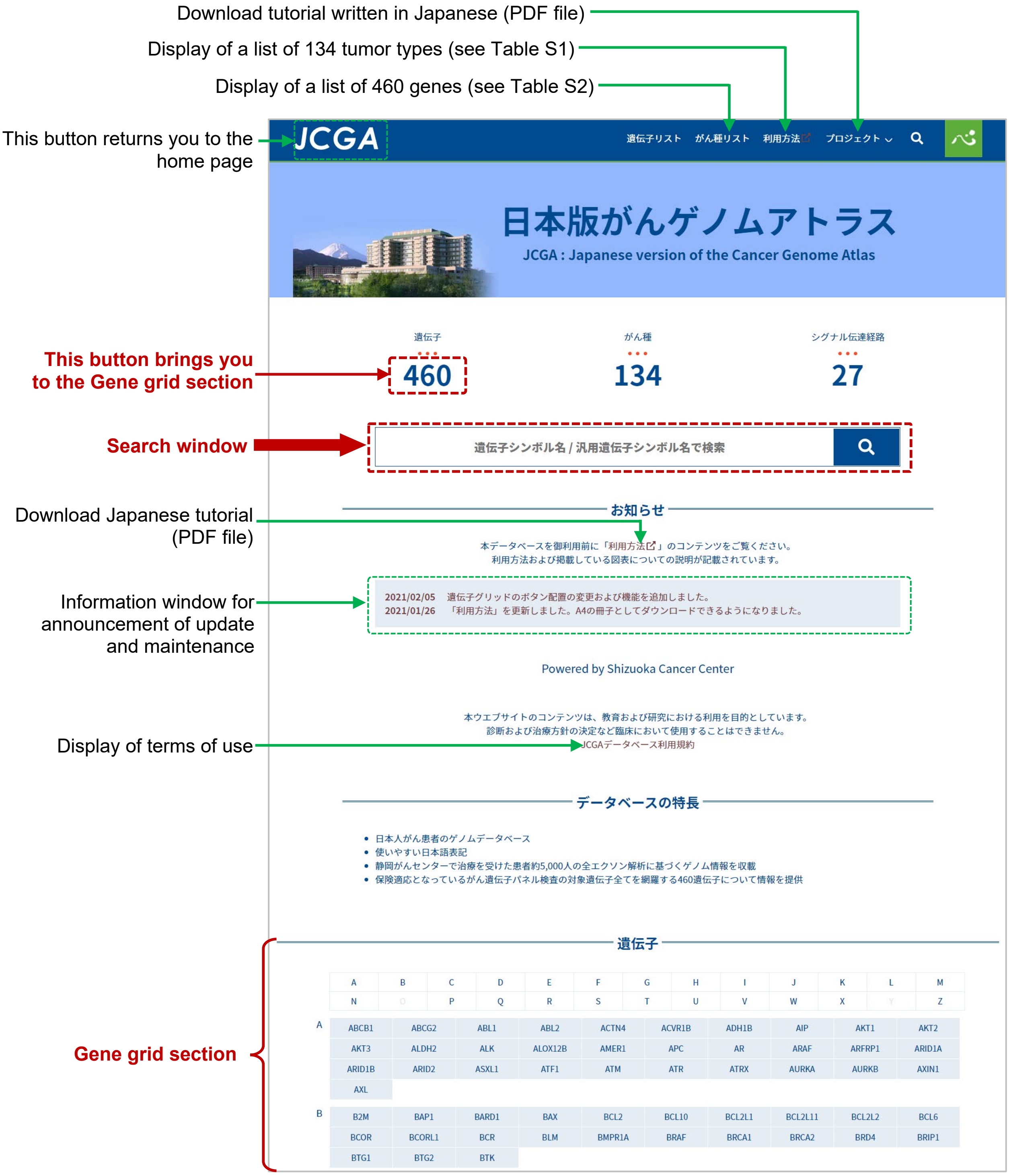

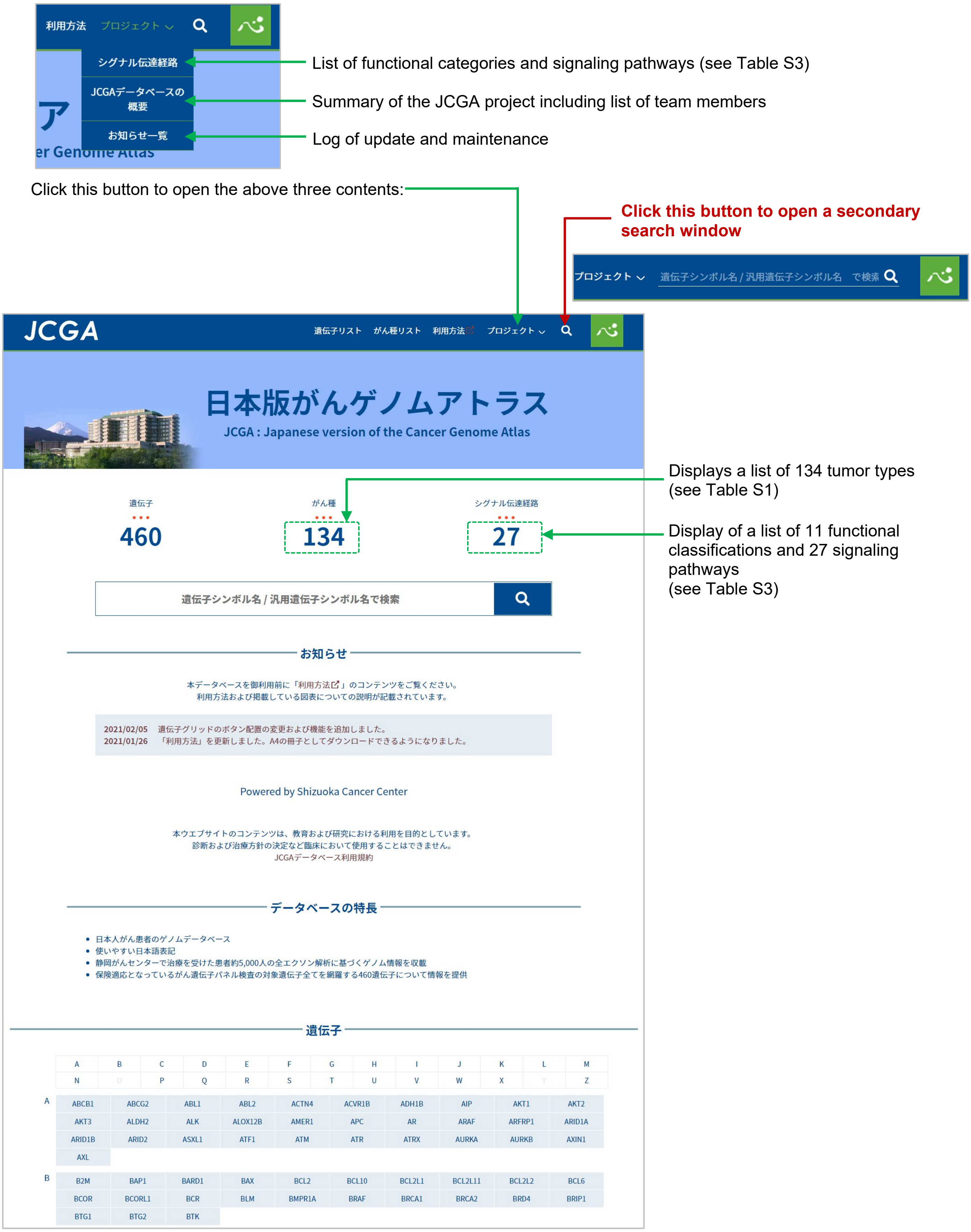
List of functional categories and signaling pathways (see Table S3)
Summary of the JCGA project including list of team members
Log of update and maintenance
Click this button to open the above three contents:
Click this button to open a secondary search window
Displays a list of 134 tumor types (see Table S1)
Display of a list of 11 functional classifications and 27 signaling pathways (see Table S3)
利用方法
プロジェクト
シグナル伝達経路
JCGAデータベースの概要
お知らせ一覧
遺伝子シンボル名 / 汎用遺伝子シンボル名 で検索
JCGA
遺伝子リスト
がん種リスト
日本版がんゲノムアトラス
JCGA : Japanese version of the Cancer Genome Atlas
遺伝子
460
がん種
134
シグナル伝達経路
27
遺伝子シンボル名 / 汎用遺伝子シンボル名で検索
お知らせ
本データベースを御利用前に「利用方法」のコンテンツをご覧ください。
利用方法および掲載している図表についての説明が記載されています。
2021/02/05 遺伝子グリッドのボタン配置の変更および機能を追加しました。
2021/01/26 「利用方法」を更新しました。A4の冊子としてダウンロードできるようになりました。
Powered by Shizuoka Cancer Center
本ウエブサイトのコンテンツは、教育および研究における利用を目的としています。
診断および治療方針の決定など臨床において使用することはできません。
JCGAデータベース利用規約
データベースの特長
日本人がん患者のゲノムデータベース
使いやすい日本語表記
静岡がんセンターで治療を受けた患者約5,000人の全エクソン解析に基づくゲノム情報を収載
保険適応となっているがん遺伝子パネル検査の対象遺伝子全てを網羅する460遺伝子について情報を提供
遺伝子

2. The resulting view of each gene page from a keyword search

1) Basic information

- Basic information includes 11 sections, such as links to relevant bioinformatics resources and gene map.

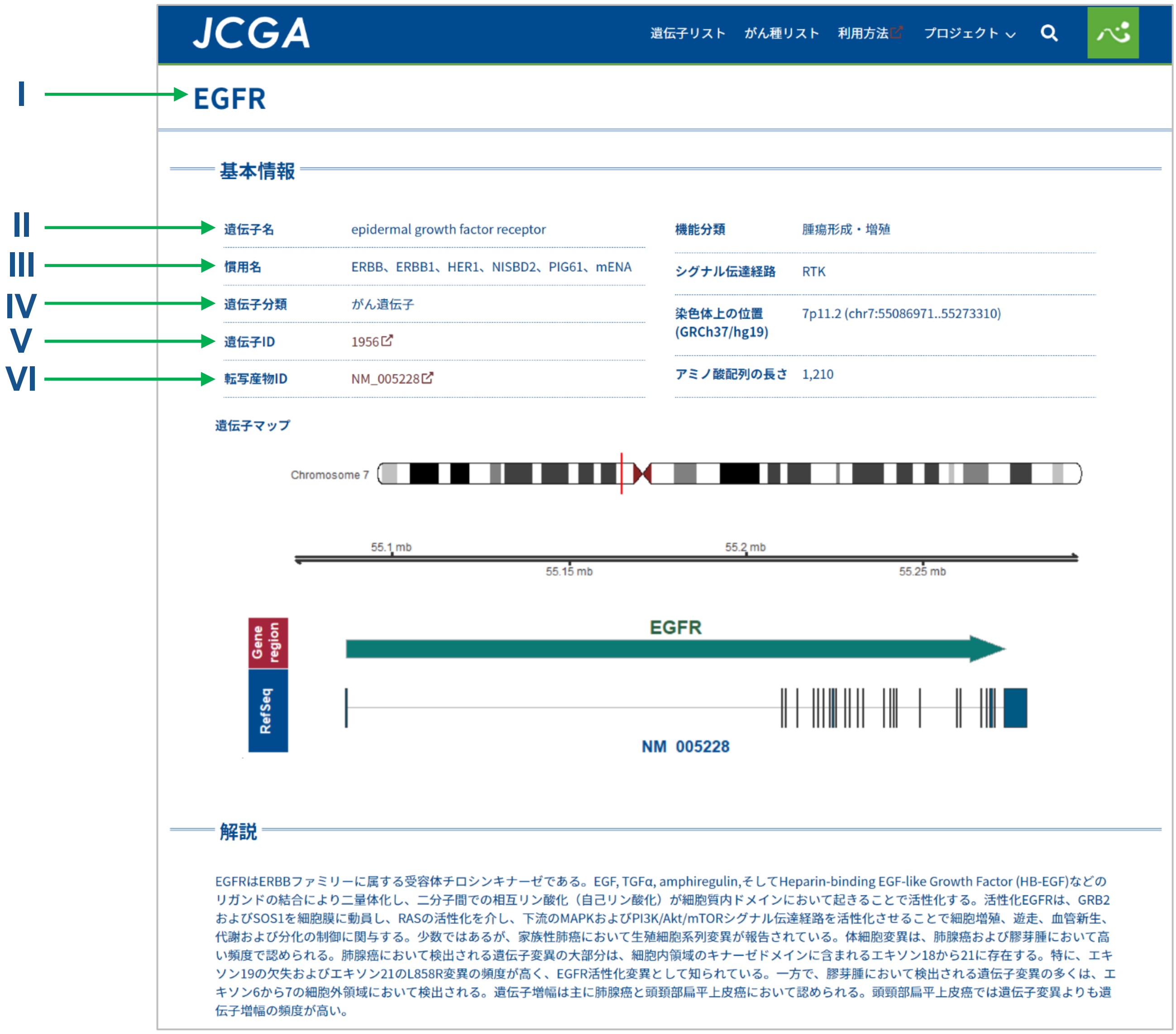

I. **Official gene symbol:** Gene symbol registered in the Human Genome Organization Gene Nomenclature Committee (HGNC) at the end of September 2020.

II. **Official gene name:** Gene name registered in HGNC at the end of September 2020.

III. **Conventional gene symbol:** Conventionally used gene symbols (e.g., HER2, MLL, MLL2, FAM123B)

IV. **Classification:** Genes are classified into following 4 categories, oncogene, tumor suppressor gene, both (oncogene / tumor suppressor gene) or --- (not assigned).

V. **Official gene ID:** Entrez ID and link to each corresponding page in the NCBI Gene database.

VI. **Transcript ID:** RefSeq transcript ID used to describe mutations in JCGA and link them to each corresponding page in RefSeq.

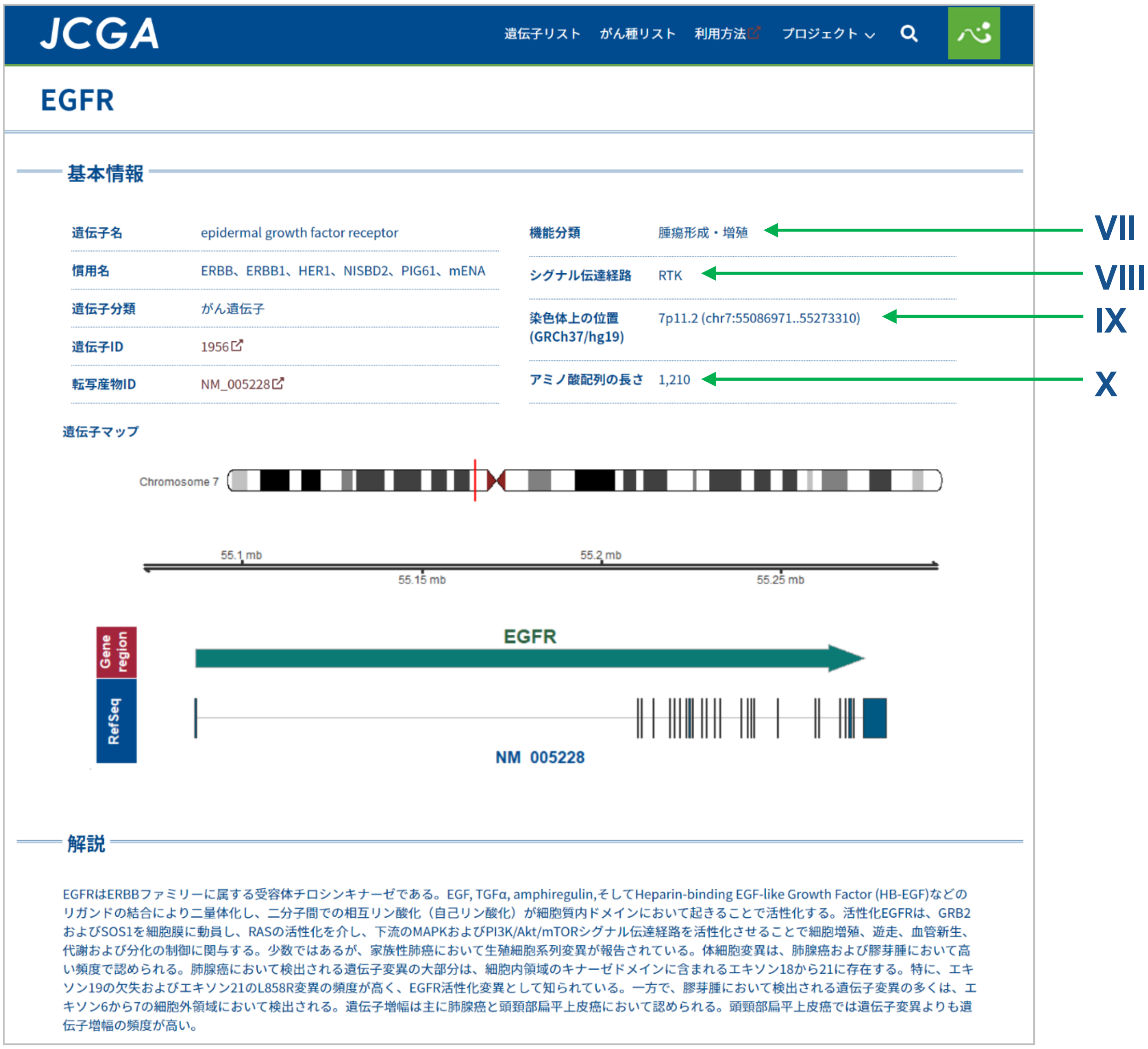

VII. Functional classification: Genes are classified into 11 functional categories. (Table S3).

VIII. Signaling pathway: Genes are classified into 27 signaling pathways. (Table S3).

IX. Chromosomal location: Cytoband and chromosomal position of 5' and 3' ends of coding region (CDS) corresponding to the indicated RefSeq transcript ID.

X. Protein length: Product length corresponding to the indicated RefSeq transcript ID. Protein product lengths are in amino acid units and do not include stop codons.

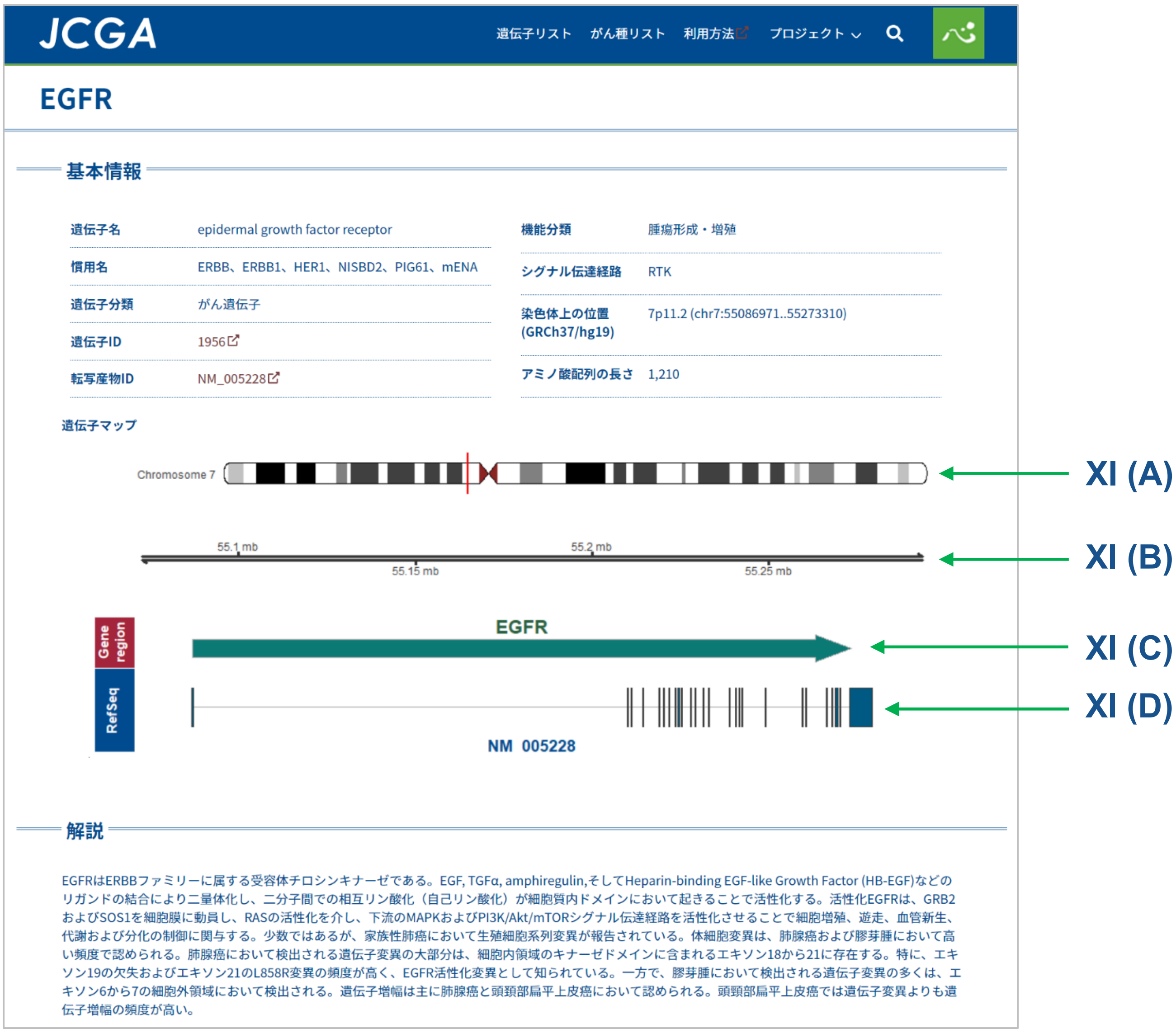

XI. Gene map: Gene map content was visualized using the R package Gviz (v. 1.34.0).

- **A) Chromosome ideogram:** The location of the selected gene is indicated on the chromosome by a pink bar.
- **B) Genomic axis:** Genomic coordinates of selected genes corresponding to highlighted regions in the chromosome ideogram.
- **C) End of coding region and direction:** Chromosomal position and direction between the 5' and 3' ends of coding region.
- **D) Location of exons:** Exon-intron organization corresponding to the indicated RefSeq transcript ID.

2) Gene summary

- Description to explain the biological functions of selected genes and their associations with carcinogenesis and tumor progression.

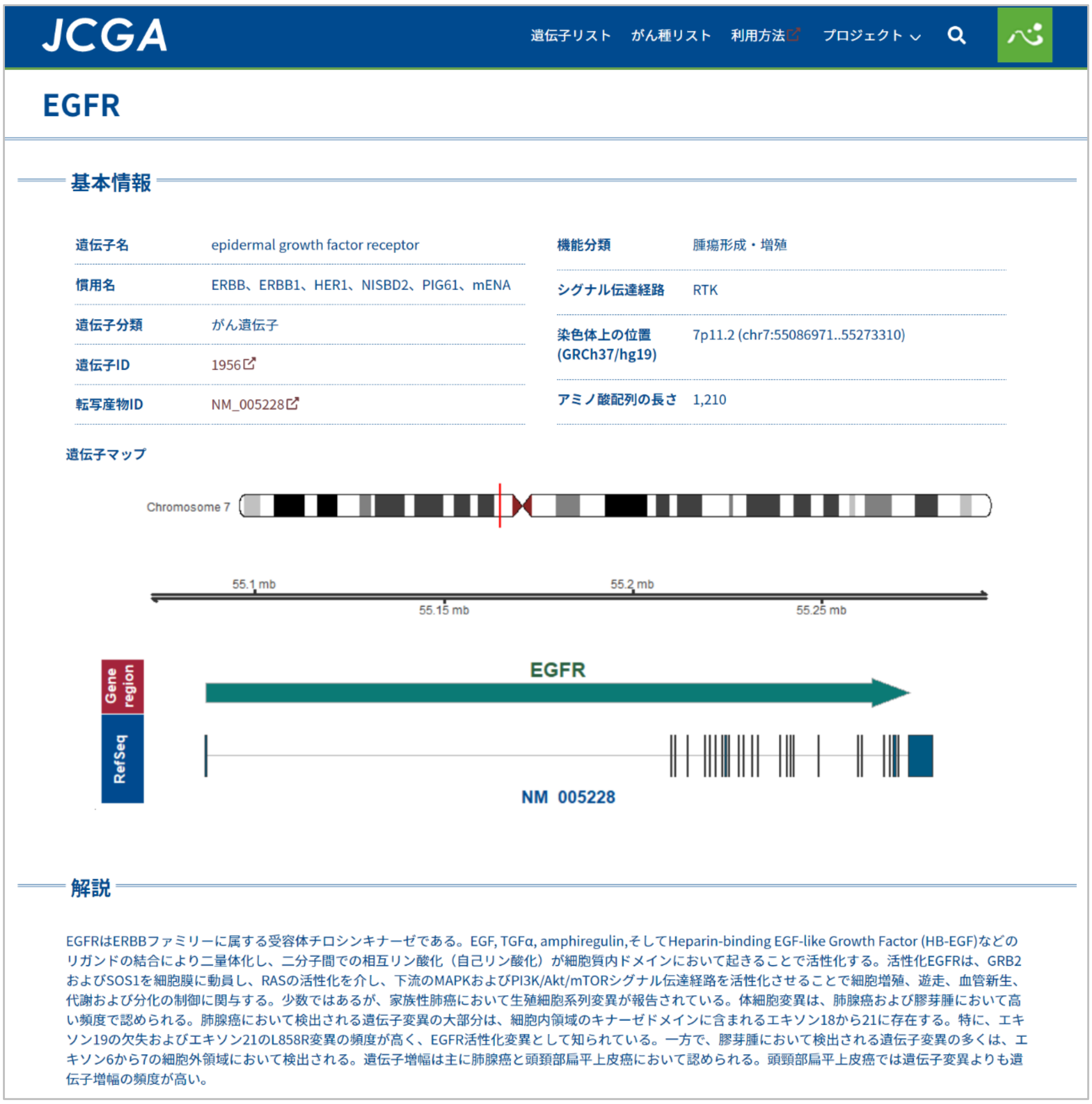

JCGA　遺伝子リスト　がん種リスト　利用方法　プロジェクト

EGFR

基本情報

遺伝子名	epidermal growth factor receptor	機能分類	腫瘍形成・増殖
慣用名	ERBB、ERBB1、HER1、NISBD2、PIG61、mENA	シグナル伝達経路	RTK
遺伝子分類	がん遺伝子	染色体上の位置 (GRCh37/hg19)	7p11.2 (chr7:55086971..55273310)
遺伝子ID	1956	アミノ酸配列の長さ	1,210
転写産物ID	NM_005228		

遺伝子マップ

解説

EGFRはERBBファミリーに属する受容体チロシンキナーゼである。EGF, TGFα, amphiregulin,そしてHeparin-binding EGF-like Growth Factor (HB-EGF)などのリガンドの結合により二量体化し、二分子間での相互リン酸化（自己リン酸化）が細胞質内ドメインにおいて起きることで活性化する。活性化EGFRは、GRB2およびSOS1を細胞膜に動員し、RASの活性化を介し、下流のMAPKおよびPI3K/Akt/mTORシグナル伝達経路を活性化させることで細胞増殖、遊走、血管新生、代謝および分化の制御に関与する。少数ではあるが、家族性肺癌において生殖細胞系列変異が報告されている。体細胞変異は、肺腺癌および膠芽腫において高い頻度で認められる。肺腺癌において検出される遺伝子変異の大部分は、細胞内領域のキナーゼドメインに含まれるエキソン18から21に存在する。特に、エキソン19の欠失およびエキソン21のL858R変異の頻度が高く、EGFR活性化変異として知られている。一方で、膠芽腫において検出される遺伝子変異の多くは、エキソン6から7の細胞外領域において検出される。遺伝子増幅は主に肺腺癌と頭頸部扁平上皮癌において認められる。頭頸部扁平上皮癌では遺伝子変異よりも遺伝子増幅の頻度が高い。

Gene summary

3) List of Pharmaceuticals and Medical Devices Agency (PMDA)-approved drugs targeting the selected gene

- PMDA-approved molecular-targeted drugs targeting the selected gene are listed.
- For drugs with multiple target genes, each drug name is described on the pages for all the applicable genes (e.g., afatinib and dacomitinib are listed in EGFR, ERBB2, and ERBB4).
- For the detailed approval of each drug, it is necessary to confirm the information published by the PMDA. JCGA provides links to the corresponding page for each drug in the PMDA.

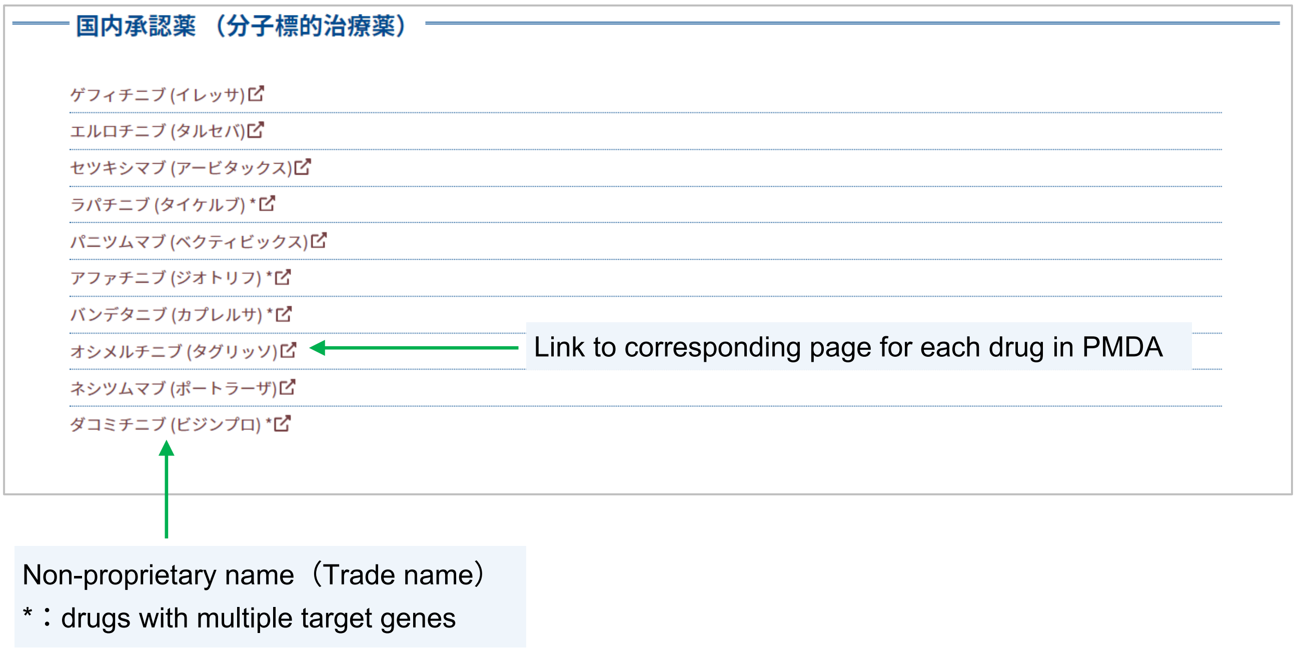

4) Pathway map

- Pathway map showing associations between cancer signaling pathways and the selected gene.
- Eight pathways, including receptor tyrosine kinase (RTK), MAPK, PI3K/Akt/mTOR, p53, apoptosis, TGF-β, WNT, and cell cycle are described.

The selected gene is highlighted with a black frame.

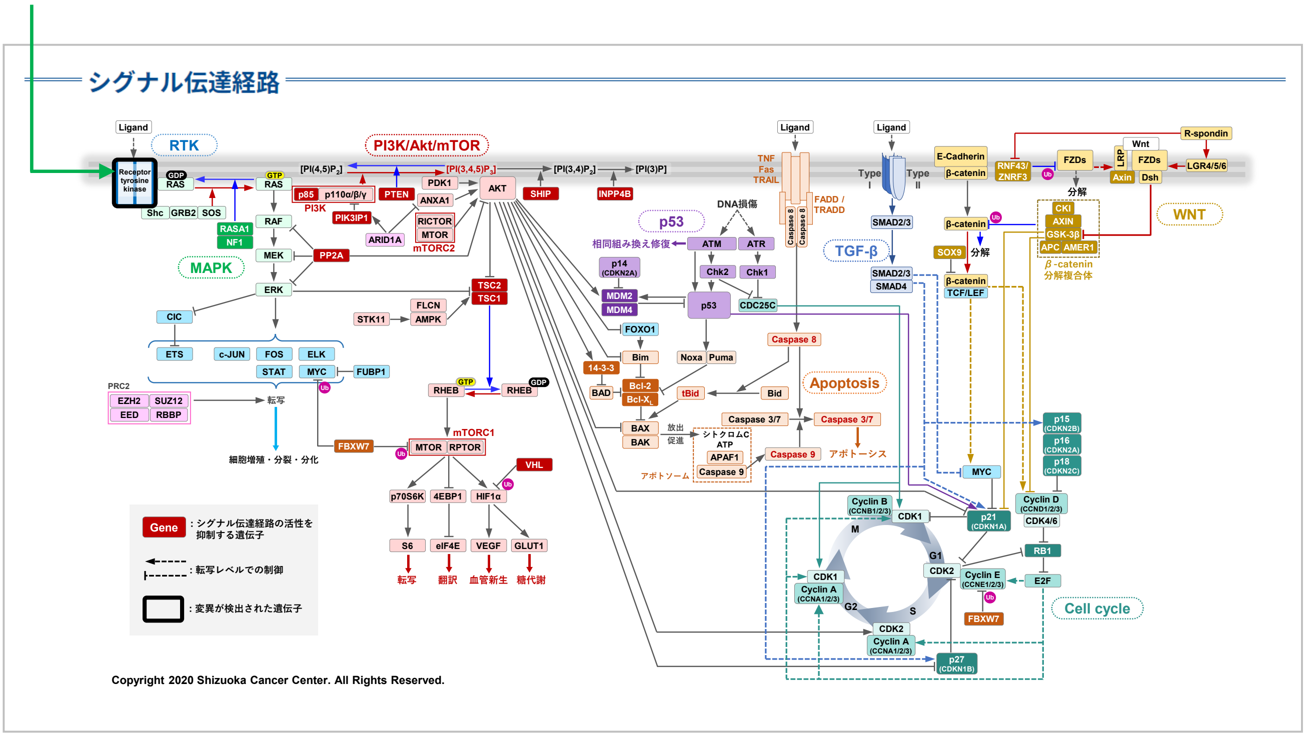

5) Graphs indicating frequency of somatic gene alterations in 30 principal tumor types

- Two graphs indicate the frequency of somatic mutations (above plot) and somatic copy number alterations (below plot) of selected genes in the 30 principal tumor types.
- Thirty principal tumor types are listed in Supplementary Table S1.

Frequency of somatic mutations (base substitutions, insertions, and deletions)

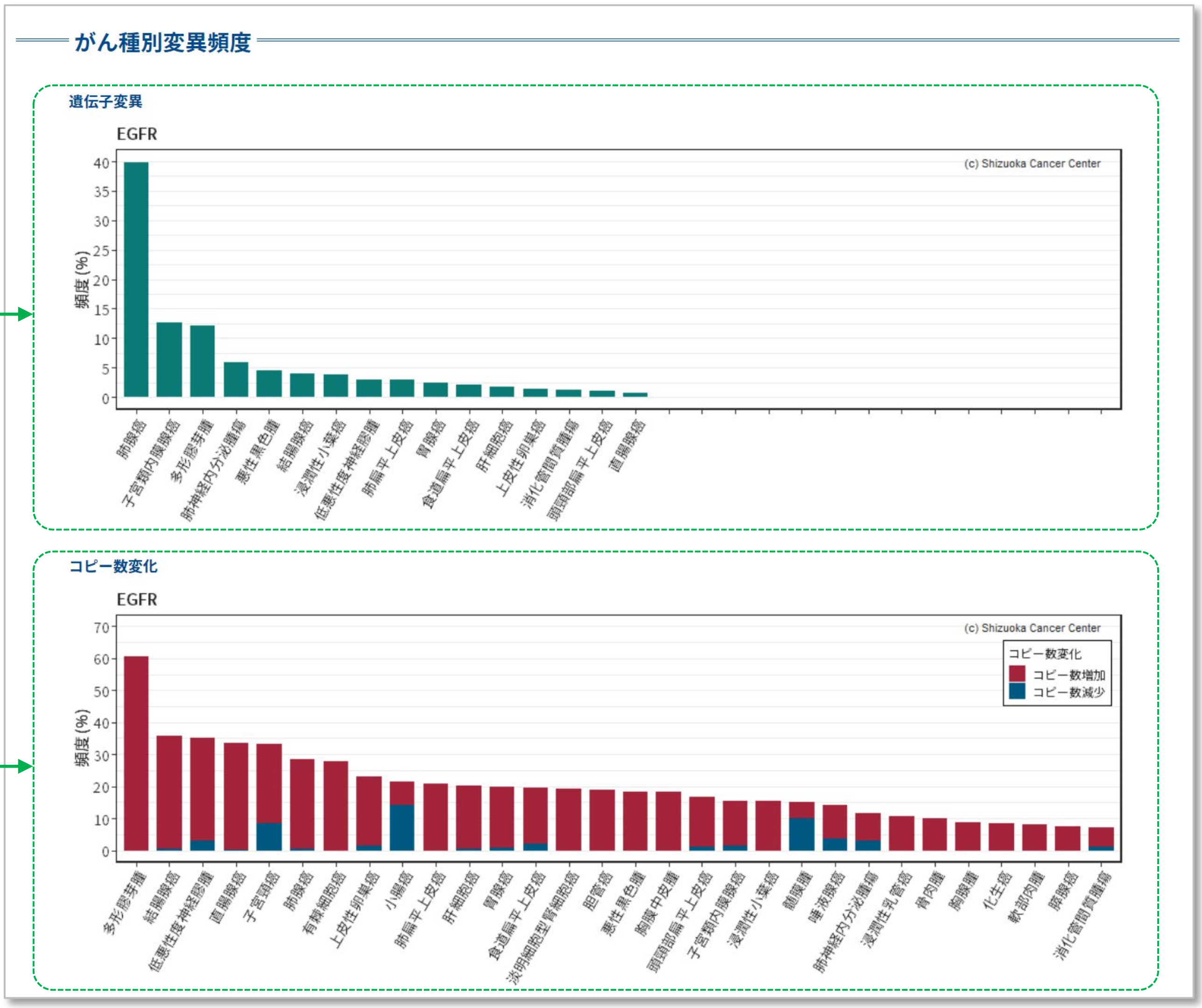

Frequency of somatic copy number alterations

Copy number gain: (red)

Copy number loss: (blue)

6) Distribution of tumor mutation burden in 30 principal tumor types

- Tumor mutation burden (TMB) was defined as the total number of somatic mutations, including base substitutions, insertions, and deletions per megabase of the effective sequence length (20× or higher coverage in each sample).
- Graphs indicating distribution of TMB in each tumor type are sorted in the ascending order of median value of TMB described in Table S4. The highest is lung neuroendocrine tumor, followed by lung squamous cell carcinoma, hepatocellular carcinoma, and colon adenocarcinoma.
- Each dot represents the TMB value in each sample and is sorted in the ascending order of TMB. The pink and navy dots indicate the TMB values of samples with and without mutations in the selected gene, respectively.
- This distribution of TMB can contribute to the simultaneous evaluation of tumor types in which patients harboring mutations in selected genes are more frequently observed and the effects of mutations in selected genes on the amount of TMB.

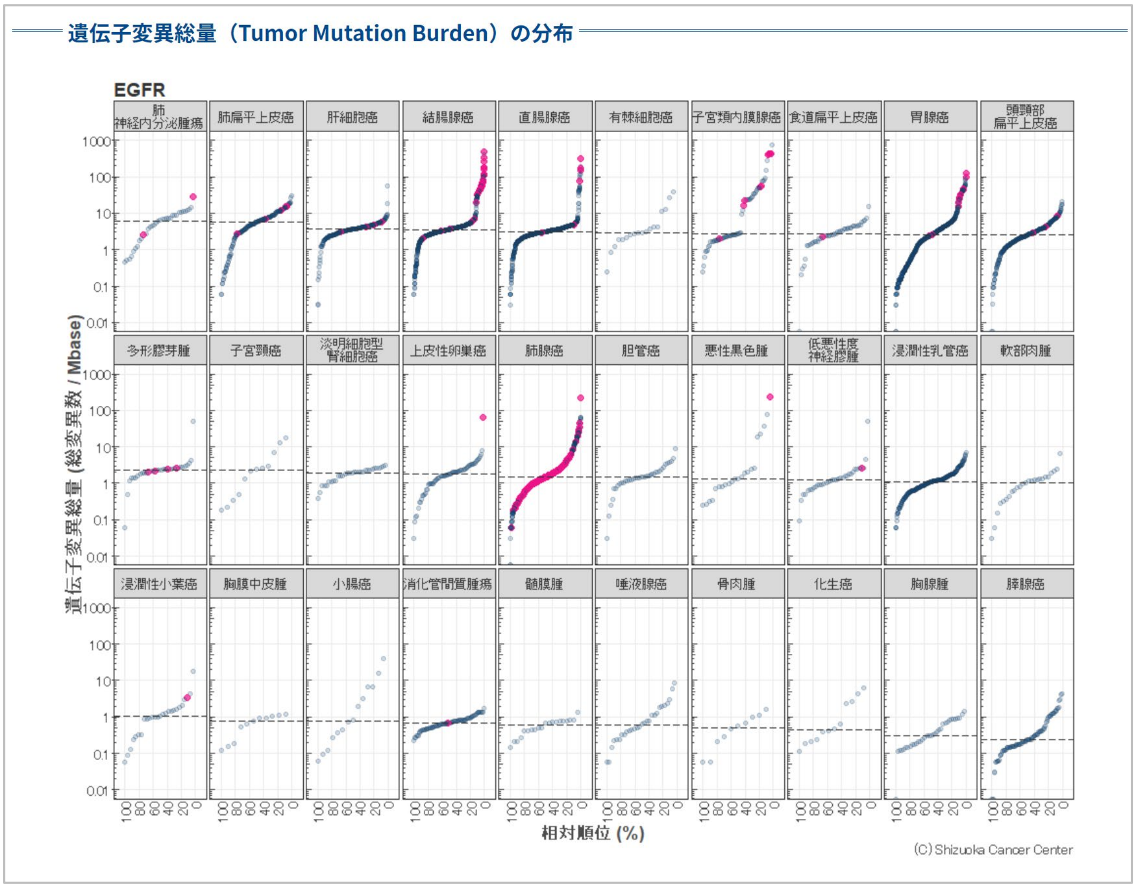

●: TMB value of samples with mutations in selected gene

●: TMB value of samples without mutations in selected gene

---: Median value of TMB in each tumor type
(see Supplementary Table S4)

Distribution density of samples

low high

7) Plots for distribution of mutations on linear protein sequence

- The lollipop plot shows the distribution of mutations (base substitutions, insertions and deletions) in the linear protein sequence.
- The lollipop plot can be used to identify putative functional mutations located in functional domains and hotspots, which are recurrently mutated positions frequently observed in oncogenes.
- The “comparison of lollipop plots” function can be used on the JCGA to compare the distribution of detected mutations in the linear protein sequence between all tumor types (above plot) and selected tumor type (below plot). This function contributes to identifying tumor types in which mutations located on hotspots or functional domains are more frequently observed.
- Mutations located in splice donor/acceptor site are not shown in these figures.

Distribution of mutations detected in all tumor types

Distribution of mutations detected in the selected tumor type

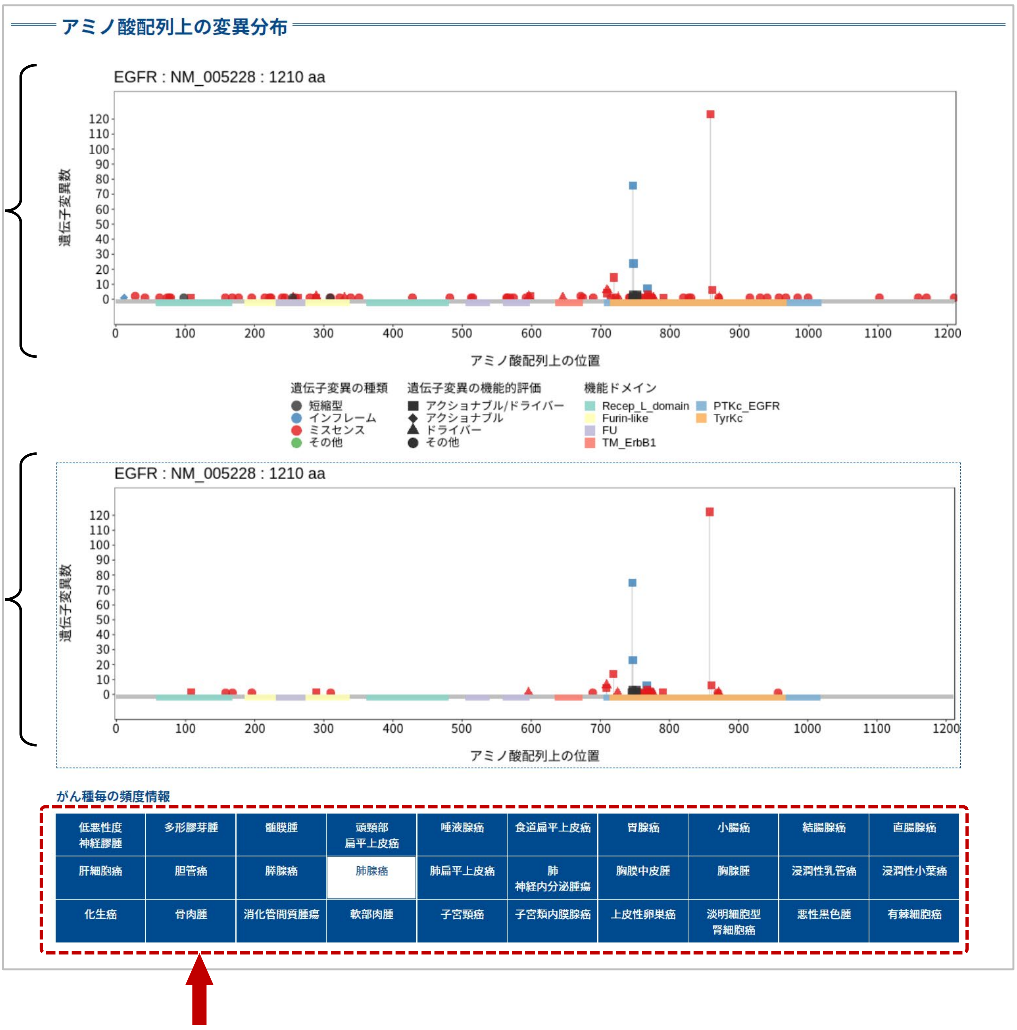

Clickable tumor type list
(Lung adenocarcinoma is selected in this example)

8) List of driver somatic mutations

■ Pathogenic mutations (Tier 1) and likely pathogenic mutations (Tier 2) defined in Nagashima *et al*. are listed in this table.

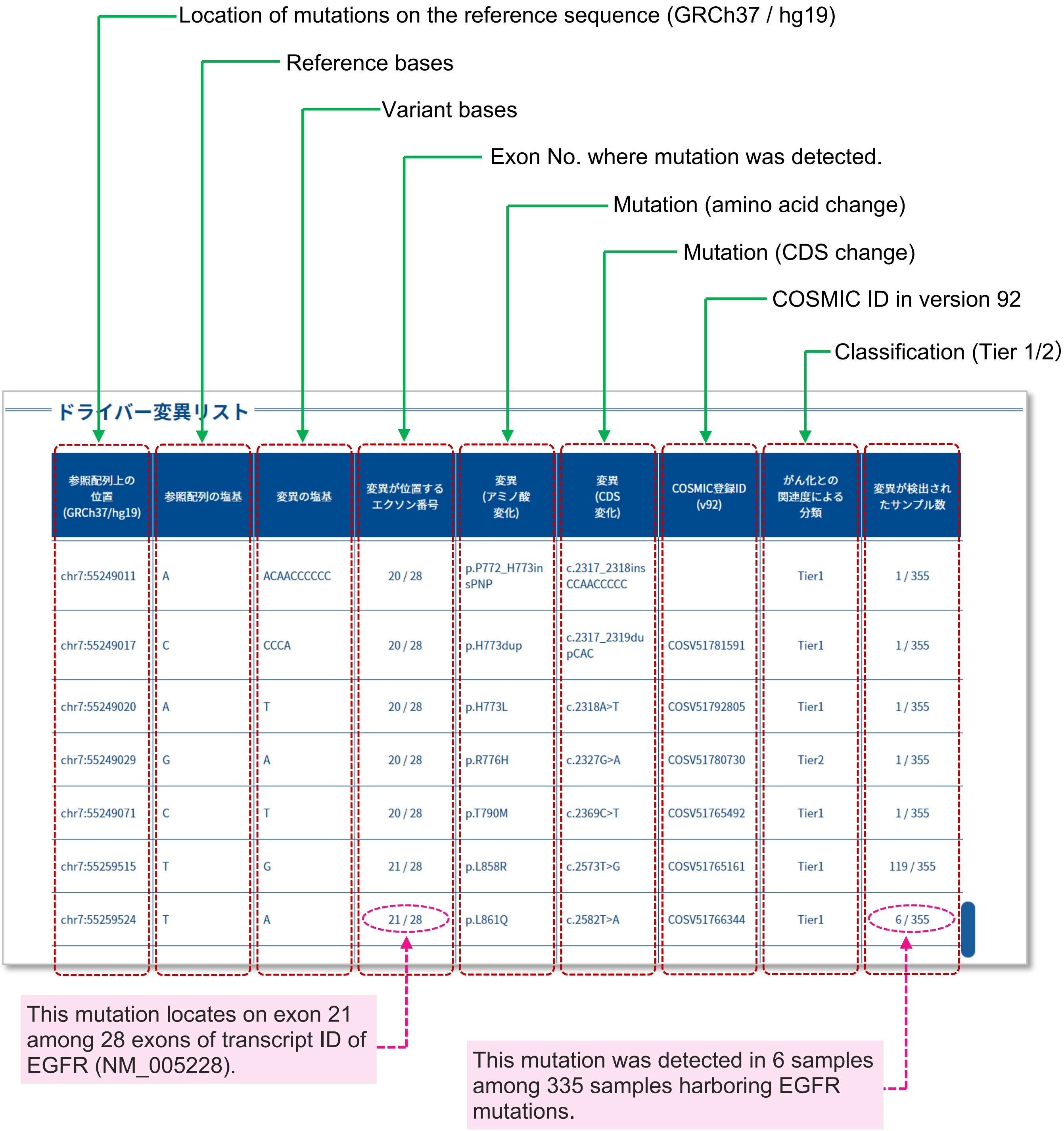

ドライバー変異リスト

参照配列上の位置 (GRCh37/hg19)	参照配列の塩基	変異の塩基	変異が位置するエクソン番号	変異 (アミノ酸変化)	変異 (CDS変化)	COSMIC登録ID (v92)	がん化との関連度による分類	変異が検出されたサンプル数
chr7:55249011	A	ACAACCCCCC	20 / 28	p.P772_H773insPNP	c.2317_2318insCCAACCCCC		Tier1	1 / 355
chr7:55249017	C	CCCA	20 / 28	p.H773dup	c.2317_2319dupCAC	COSV51781591	Tier1	1 / 355
chr7:55249020	A	T	20 / 28	p.H773L	c.2318A>T	COSV51792805	Tier1	1 / 355
chr7:55249029	G	A	20 / 28	p.R776H	c.2327G>A	COSV51780730	Tier2	1 / 355
chr7:55249071	C	T	20 / 28	p.T790M	c.2369C>T	COSV51765492	Tier1	1 / 355
chr7:55259515	T	G	21 / 28	p.L858R	c.2573T>G	COSV51765161	Tier1	119 / 355
chr7:55259524	T	A	21 / 28	p.L861Q	c.2582T>A	COSV51766344	Tier1	6 / 355

Nagashima T, Yamaguchi K, Urakami K, Shimoda Y, Ohnami S, Ohshima K et al. Japanese version of The Cancer Genome Atlas, JCGA, established using fresh frozen tumors obtained from 5143 cancer patients. *Cancer Sci* 2020; **111**: 687-699.
