# Supplementary material for: JCGA: the Japanese version of the Cancer Genome Atlas and its contribution to the interpretation of gene alterations detected in clinical cancer genome sequencing
Source: Hum Genome Var. 2021 Sep 30;8:38. doi: 10.1038/s41439-021-00170-w (PMC8481308; doi:10.1038/s41439-021-00170-w)
Supplement: Supplementary file 2 — Supplementary Table S1. Tumor types in JCGA (134) classified according to Oncotree and/or The Cancer Genome Atlas (TCGA) criteria. [file 41439_2021_170_MOESM2_ESM.pdf]

Supplementary Table S1. Tumor types in JCGA (134) classified according to Oncotree and/or The Cancer Genome Atlas (TCGA) criteria.

Tumor site	Tumor type	Oncotree[1] / TCGA[2] code	Classification criteria	No. of samples	Principal tumor types in JCGA	Tumor types selected for study in TCGA
Central nervous system/Brain	Lower-Grade Glioma [3]	LGG	TCGA	34	✓	✓
	Glioblastoma Multiforme	GBM	Oncotree	33	✓	✓
	Meningioma	MNG	Oncotree	20	✓	
	Malignant Lymphoma	MLYM	Oncotree	3		
	Desmoplastic Small-Round-Cell Tumor	DSRCT	Oncotree	1		
Eye	Uveal Melanoma	UM	Oncotree	2		✓
Head and Neck	Head and Neck Squamous Cell Carcinoma	HNSC	Oncotree	288	✓	✓
	Salivary Carcinoma	SACA	Oncotree	28	✓	
	Adenosquamous Carcinoma of the Tongue	ASCT	Oncotree	1		
	Head and Neck Neuroendocrine Carcinoma	HNNE	Oncotree	1		
	Head and Neck Mucosal Melanoma	HNMUCM	Oncotree	1		
	Spindle Cell Carcinoma of the Head and Neck	---	---	1		
	Head and Neck Carcinoma, Other	OHNCA	Oncotree	8		
Esophagus	Esophageal Squamous Cell Carcinoma	ESCC	Oncotree	46	✓	✓
	Esophageal Adenocarcinoma	ESCA	Oncotree	6		
	Esophageal Carcinosarcoma	---	---	2		
Stomach	Stomach Adenocarcinoma [4]	STAD	TCGA	574	✓	✓
	Adenosquamous Carcinoma of the Stomach	STAS	Oncotree	3		
Esophagus/Stomach	Gastrointestinal Neuroendocrine Tumors of the Esophagus/Stomach	GINETES	Oncotree	15		
	Adenocarcinoma of the Gastroesophageal Junction	GEJ	Oncotree	1		
Small intestine	Small Intestinal Carcinoma	SBC	Oncotree	14	✓	
	Small Bowel Well-Differentiated Neuroendocrine Tumor	SBWDNET	Oncotree	5		
Appendix	Mucinous Adenocarcinoma of the Appendix	MAAP	Oncotree	2		
Large intestine	Colon Adenocarcinoma	COAD	Oncotree	964	✓	✓
	Rectal Adenocarcinoma	READ	Oncotree	755	✓	✓
	Colon Cancer, NOS	---	---	1		
Anus	Anal Squamous Cell Carcinoma	ANSC	Oncotree	2		
	Anorectal Mucosal Melanoma	ARMM	Oncotree	1		
Liver	Hepatocellular Carcinoma	HCC	Oncotree	222	✓	✓
	Malignant Nonepithelial Tumor of the Liver	LIMNET	Oncotree	5		
	Hepatocellular Carcinoma plus Intrahepatic Cholangiocarcinoma	HCCIHCH	Oncotree	5		
	Angiomyolipoma	---	---	2		
	Liver Angiosarcoma	LIAS	Oncotree	1		
Biliary tract	Cholangiocarcinoma	CHOL	Oncotree	37	✓	✓
	Gallbladder Cancer	GBC	Oncotree	5		
	Neuroendocrine Carcinoma	---	---	1		
Ampulla of Vater	Ampullary Carcinoma	AMPCA	Oncotree	3		
Pancreas	Pancreatic Adenocarcinoma	PAAD	Oncotree	95	✓	✓
	Pancreatic Neuroendocrine Tumor	PANET	Oncotree	11		
	Adenosquamous Carcinoma of the Pancreas	PAASC	Oncotree	10		
	Intraductal Papillary Mucinous Neoplasm	IPMN	Oncotree	7		
	Solid Pseudopapillary Neoplasm of the Pancreas	SPN	Oncotree	3		
	Undifferentiated Carcinoma of the Pancreas	UCP	Oncotree	1		
Lung	Lung Adenocarcinoma	LUAD	Oncotree	644	✓	✓
	Lung Squamous Cell Carcinoma	LUSC	Oncotree	173	✓	✓
	Lung Neuroendocrine Tumor	LNET	Oncotree	34	✓	
	Lung Adenosquamous Carcinoma	LUAS	Oncotree	21		
	Pleomorphic Carcinoma of the Lung	LUPC	Oncotree	9		
	Poorly Differentiated Non-Small Cell Lung Cancer	NSCLCPD	Oncotree	2		
	Adenoid Cystic Carcinoma of the Lung	LUACC	Oncotree	2		
	Large Cell Lung Carcinoma	LCLC	Oncotree	2		
	Sclerosing Pneumocytoma	---	---	1		
	Mucinous AIS-like Bronchiolar Cell Hyperplasia	---	---	1		
	Mixed Squamous Cell and Glandular Papilloma	---	---	1		

Supplementary Table S1. Continued

Tumor site	Tumor type	Oncotree / TCGA code	Classification criteria	No. of Samples	Principal tumor types in JCGA	Tumor types selected in TCGA
Pleura	Pleural Mesothelioma	PLMESO	Oncotree	11	✓	✓
Mediastinum	Mediastinal Non-seminomatous Germ Cell Tumor	---	---	2		
	Thymic MALT Lymphoma	---	---	1		
	Mediastinal Tumor, NOS	---	---	1		
Thymus	Thymoma	THYM	Oncotree	34	✓	✓
	Thymic Carcinoma	THYC	Oncotree	7		
Breast	Breast Invasive Ductal Carcinoma	IDC	Oncotree	235	✓	✓
	Breast Invasive Lobular Carcinoma	ILC	Oncotree	26	✓	✓
	Metaplastic Breast Cancer	MBC	Oncotree	12	✓	
	Breast Invasive Mixed Mucinous Carcinoma	IMMC	Oncotree	7		
	Breast Ductal Carcinoma In Situ	DCIS	Oncotree	3		
	Breast Mixed Ductal and Lobular Carcinoma	MDLC	Oncotree	2		
	Phyllodes Tumor of the Breast	PT	Oncotree	1		
Thyroid	Papillary Thyroid Cancer	THPA	Oncotree	3		✓
	Anaplastic Thyroid Cancer	THAP	Oncotree	2		
	Medullary Thyroid Cancer	THME	Oncotree	1		
Bone	Osteosarcoma	OS	Oncotree	10	✓	
	Chondrosarcoma	CHS	Oncotree	2		
	Ewing Sarcoma	ES	Oncotree	1		
	Osteochondroma	---	---	1		
	Bone Tumor, NOS	---	---	1		
Soft tissue	Gastrointestinal Stromal Tumor	GIST	Oncotree	83	✓	
	Liposarcoma	LIPO	Oncotree	5		
	Rhabdomyosarcoma	RMS	Oncotree	2		
	Solitary Fibrous Tumor/Hemangiopericytoma	SFT	Oncotree	1		
	Well-Differentiated Liposarcoma	WDLS	Oncotree	1		
	Low-Grade Fibromyxoid Sarcoma	LGFMS	Oncotree	1		
	Soft Tissue Myoepithelial Carcinoma	STMYEC	Oncotree	1		
	Paraganglioma	PGNG	Oncotree	1		✓
	Spindle Cell/Sclerosing Rhabdomyosarcoma	SCSRMS	Oncotree	1		
	Epithelioid Hemangioendothelioma	EHAE	Oncotree	1		
	Sarcoma, NOS	SARCNOS	Oncotree	3		
Soft tissue/Peripheral nervous system	Sarcoma [5]	SARC	TCGA	25	✓	✓
Cervix	Cervical Squamous Cell Carcinoma and Endocervical Adenocarcinoma [6]	CESC	TCGA	12	✓	✓
	Cervical Neuroendocrine Tumor	CENE	Oncotree	1		
Uterus	Uterine Endometrioid Carcinoma	UEC	Oncotree	71	✓	✓
	Uterine Carcinosarcoma/Uterine Malignant Mixed Mullerian Tumor	UCS	Oncotree	9		✓
	Uterine Serous Carcinoma/Uterine Papillary Serous Carcinoma	USC	Oncotree	6		
	Uterine Mixed Endometrial Carcinoma	UMEC	Oncotree	2		
	Uterine Adenosarcoma	UAS	Oncotree	2		
	Uterine Clear Cell Carcinoma	UCCC	Oncotree	2		
	Uterine Perivascular Epithelioid Cell Tumor	UPECOMA	Oncotree	1		
	Uterine Neuroendocrine Carcinoma	UNEC	Oncotree	1		
Ovary/Fallopian tube	Ovarian Epithelial Tumor	OVT	Oncotree	69	✓	✓
	Immature Teratoma	OIMT	Oncotree	2		
	Struma Ovarii	---	---	2		
	High-Grade Serous Fallopian Tube Cancer	HGSFT	Oncotree	1		
	Sex Cord Stromal Tumor	SCST	Oncotree	1		
	Mixed Germ Cell Tumor	OMGCT	Oncotree	1		
	Yolk Sac Tumor	OYST	Oncotree	1		
	Struma Ovarii + Dermoid Cyst	---	---	1		
	Adult Granulosa Cell Tumor	---	---	1		
	Dedifferentiated Carcinoma of the Ovary	---	---	1		
	Squamous Cell Carcinoma of the Ovary	---	---	1		
	Ovarian Cancer, NOS	---	---	1		

Supplementary Table S1. Continued

Tumor site	Tumor type	Oncotree / TCGA code	Classification criteria	No. of Samples	Principal tumor types in JCGA	Tumor types selected in TCGA
Peritoneum	Peritoneal Serous Carcinoma	PSEC	Oncotree	2		
	Teratoma of the Retroperitoneum	---	---	1		
Ovary/Fallopian tube + Uterus	Clear Cell Ovarian Cancer + Uterine Endometrioid Carcinoma	CCOV + UEC	Oncotree	1		
Kidney	Renal Clear Cell Carcinoma	CCRCC	Oncotree	31	✓	✓
	Chromophobe Renal Cell Carcinoma	CHRCC	Oncotree	2		✓
	Papillary Renal Cell Carcinoma	PRCC	Oncotree	2		✓
	Renal Neuroendocrine Tumor	RNET	Oncotree	1		
	Juxtaglomerular Cell Tumor	---	---	1		
Bladder/Urinary tract	Upper Tract Urothelial Carcinoma	UTUC	Oncotree	2		✓
Skin	Melanoma	MEL	Oncotree	22	✓	✓
	Cutaneous Squamous Cell Carcinoma	CSCC	Oncotree	18	✓	
	Dermatofibrosarcoma Protuberans	DFSP	Oncotree	6		
	Extramammary Paget Disease	EMPD	Oncotree	5		
	Poroma/Acrospiroma	PORO	Oncotree	2		
	Merkel Cell Carcinoma	MCC	Oncotree	1		
	Porocarcinoma/Spiroadenocarcinoma	POCA	Oncotree	1		
	Sweat Gland Carcinoma/Apocrine Eccrine Carcinoma	AECA	Oncotree	1		
	Basal Cell Carcinoma	BCC	Oncotree	1		
Vulva/Vagina	Squamous Cell Carcinoma of the Vulva/Vagina	VSC	Oncotree	4		
	Mucosal Melanoma of the Vulva/Vagina	VMM	Oncotree	1		
	Neurofibroma	NFIB	Oncotree	2		
Peripheral nervous system	Neuroblastoma	NBL	Oncotree	1		
Head and neck/Thymus/Pancreas/Small intestine	Multiple Endocrine Neoplasia Type 1	---	---	6		
Lymphoid	Lymphoid Neoplasm	LNM	Oncotree	5		
Other	Cancer of Unknown Primary	CUP	Oncotree	1		

JCGA includes genome information of 4,907 surgically resected primary tumor samples obtained from 4,753 Japanese cancer patients.

NOS: not otherwise specified

[1] Oncotree: http://oncotree.mskcc.org/#/home

[2] TCGA (The Cancer Genome Atlas): https://gdc.cancer.gov/resources-tcga-users/tcga-code-tables/tcga-study-abbreviations

The following four tumor types conformed to TCGA criteria, including subtypes classified based on Oncotree criteria.

[3] Lower-grade glioma (n = 34) is diffusely infiltrative low-grade or intermediate-grade glioma (World Health Organization grade II or III), including the following five subtypes classified based on Oncotree criteria: anaplastic astrocytoma (AASTR; WHO grade III; n = 4), anaplastic ganglioglioma (AGNG; WHO grade III; n = 1), anaplastic oligodendroglioma (AODG; WHO grade III; n = 2), astrocytoma (ASTR; WHO grade II; n = 13), and oligodendroglioma (ODG; WHO grade II; n = 14).

[4] Stomach adenocarcinoma (n = 574), including both stomach adenocarcinoma (STAD; n=313) and undifferentiated stomach adenocarcinoma (USTAD; n = 261) classified based on Oncotree classification.

[5] Sarcoma (n = 25), including the following six subtypes and a combind subtype classified based on Oncotree criteria: dedifferentiated liposarcoma (DDLS, n = 4), leiomyosarcoma (LMS; n = 4), undifferentiated pleomorphic sarcoma/malignant fibrous histiocytoma/high-grade spindle cell sarcoma (MFH; n = 3), myxofibrosarcoma (MFS, N = 9), malignant peripheral nerve sheath tumor (MPNST; n = 3), synovial sarcoma (SYNS; n = 1), and myxofibrosarcoma + undifferentiated pleomorphic sarcoma/malignant fibrous histiocytoma/high-grade spindle cell sarcoma (MFS + MFH; n = 1).

[6] Cervical squamous cell carcinoma and endocervical adenocarcinoma (n = 12), including the following three subtypes classified based on Oncotree criteria: cervical adenocarcinoma (CEAD; n = 4), cervical adenosquamous carcinoma (CEAS; n = 2), and cervical squamous cell carcinoma (CESC, n = 6).
