# Supplementary material for: JCGA: the Japanese version of the Cancer Genome Atlas and its contribution to the interpretation of gene alterations detected in clinical cancer genome sequencing
Source: Hum Genome Var. 2021 Sep 30;8:38. doi: 10.1038/s41439-021-00170-w (PMC8481308; doi:10.1038/s41439-021-00170-w)
Supplement: Supplementary file 3 — Supplementary Table S2. Information in JCGA for 460 cancer-associated genes [file 41439_2021_170_MOESM3_ESM.pdf]

Supplementary Table S2. Information in JCGA for 460 cancer-associated genes

Official gene symbol	Official gene name	Chromosomal location	Official gene ID	RefSeq transcript ID	Classification	Functional classification	Signaling pathway
ABCB1	ATP binding cassette subfamily B member 1	7q21.12	5243	NM_000927	---	Metabolism	Drug metabolism
ABCG2	ATP binding cassette subfamily G member 2 (Junior blood group)	4q22.1	9429	NM_004827	---	Metabolism	Drug metabolism
ABL1	ABL proto-oncogene 1, non-receptor tyrosine kinase	9q34.12	25	NM_005157	Oncogene	Tumor growth and progression	RTK
ABL2	ABL proto-oncogene 2, non-receptor tyrosine kinase	1q25.2	27	NM_007314	Oncogene	Tumor growth and progression	RTK
ACTN4	actinin alpha 4	19q13.2	81	NM_004924	---	---	---
ACVR1B	activin A receptor type 1B	12q13.13	91	NM_004302	Tumor suppressor gene	Tumor growth and progression	TGF-B
ADH1B	alcohol dehydrogenase 1B (class I), beta polypeptide	4q23	125	NM_000668	---	Metabolism	Metabolic pathway
AIP	aryl hydrocarbon receptor interacting protein	11q13.2	9049	NM_003977	---	---	---
AKT1	AKT serine/threonine kinase 1	14q32.33	207	NM_005163	Oncogene	Tumor growth and progression	PI3K/Akt/mTOR
AKT2	AKT serine/threonine kinase 2	19q13.2	208	NM_001626	Oncogene	Tumor growth and progression	PI3K/Akt/mTOR
AKT3	AKT serine/threonine kinase 3	1q43-q44	10000	NM_005465	Oncogene	Tumor growth and progression	PI3K/Akt/mTOR
ALDH2	aldehyde dehydrogenase 2 family member	12q24.12	217	NM_000690	---	Metabolism	Drug metabolism
ALK	ALK receptor tyrosine kinase	2p23.2-p23.1	238	NM_004304	Oncogene	Tumor growth and progression	RTK
ALOX12B	arachidonate 12-lipoxygenase, 12R type	17p13.1	242	NM_001139	---	Immune	Immune
AMER1	APC membrane recruitment protein 1	Xq11.2	139285	NM_152424	Tumor suppressor gene	Differentiation	WNT
APC	APC regulator of WNT signaling pathway	5q22.2	324	NM_001127510	Tumor suppressor gene	Differentiation	WNT
AR	androgen receptor	Xq12	367	NM_000044	Oncogene	Tumor growth and progression	Nuclear receptor
ARAF	A-Raf proto-oncogene, serine/threonine kinase	Xp11.3	369	NM_001654	Oncogene	Tumor growth and progression	MAPK
ARFRP1	ADP ribosylation factor-related protein 1	20q13.33	10139	NM_001267547	---	---	---
ARID1A	AT-rich interaction domain 1A	1p36.11	8289	NM_006015	Tumor suppressor gene	Epigenetic regulation	Epigenetic modification
ARID1B	AT-rich interaction domain 1B	6q25.3	57492	NM_017519	Tumor suppressor gene	Epigenetic regulation	Epigenetic modification
ARID2	AT-rich interaction domain 2	12q12	196528	NM_152641	Tumor suppressor gene	Epigenetic regulation	Epigenetic modification
ASXL1	ASXL transcriptional regulator 1	20q11.21	171023	NM_015338	Tumor suppressor gene	Epigenetic regulation	Epigenetic modification
ATF1	activating transcription factor 1	12q13.12	466	NM_005171	Oncogene	Transcriptional regulation	Transcriptional regulation
ATM	ATM serine/threonine kinase	11q22.3	472	NM_000051	Tumor suppressor gene	Genome maintenance	TP53
ATR	ATR serine/threonine kinase	3q23	545	NM_001184	Tumor suppressor gene	Genome maintenance	TP53
ATRX	ATRX chromatin remodeler	Xq21.1	546	NM_000489	Tumor suppressor gene	Epigenetic regulation	Epigenetic modification
AURKA	aurora kinase A	20q13.2	6790	NM_198433	Oncogene	Cell cycle	Cell division
AURKB	aurora kinase B	17p13.1	9212	NM_004217	Oncogene	Cell cycle	Cell division
AXIN1	axin 1	16p13.3	8312	NM_003502	Tumor suppressor gene	Differentiation	WNT
AXL	AXL receptor tyrosine kinase	19q13.2	558	NM_021913	Oncogene	Tumor growth and progression	RTK
B2M	beta-2-microglobulin	15q21.1	567	NM_004048	Tumor suppressor gene	Immune	Immune
BAP1	BRCA1 associated protein 1	3p21.1	8314	NM_004656	Tumor suppressor gene	Protein homeostasis	Protein homeostasis
BARD1	BRCA1 associated RING domain 1	2q35	580	NM_000465	Tumor suppressor gene	Genome maintenance	Core DNA Damage Response
BAX	BCL2 associated X, apoptosis regulator	19q13.33	581	NM_138761	Tumor suppressor gene	Cell death	Apoptosis
BCL10	BCL10 immune signaling adaptor	1p22.3	8915	NM_003921	Tumor suppressor gene	Tumor growth and progression	NFKB
BCL2	BCL2 apoptosis regulator	18q21.33	596	NM_000633	Oncogene	Cell death	Apoptosis
BCL2L1	BCL2-like 1	20q11.21	598	NM_138578	Oncogene	Cell death	Apoptosis
BCL2L11	BCL2-like 11	2q13	10018	NM_138621	Tumor suppressor gene	Cell death	Apoptosis
BCL2L2	BCL2-like 2	14q11.2	599	NM_004050	---	Cell death	Apoptosis
BCL6	BCL6 transcription repressor	3q27.3	604	NM_001130845	Oncogene	Immune	Immune
BCOR	BCL6 corepressor	Xp11.4	54880	NM_001123385	Tumor suppressor gene	Epigenetic regulation	Epigenetic modification
BCORL1	BCL6 corepressor-like 1	Xq26.1	63035	NM_021946	Oncogene / Tumor suppressor gene	Epigenetic regulation	Epigenetic modification
BCR	BCR activator of RhoGEF and GTPase	22q11.23	613	NM_004327	Oncogene	---	---
BLM	BLM RecQ-like helicase	15q26.1	641	NM_001287246	Tumor suppressor gene	Genome maintenance	Core DNA Damage Response
BMPR1A	bone morphogenetic protein receptor type 1A	10q23.2	657	NM_004329	Oncogene / Tumor suppressor gene	Tumor growth and progression	TGF-B
BRAF	B-Raf proto-oncogene, serine/threonine kinase	7q34	673	NM_004333	Oncogene	Tumor growth and progression	MAPK
BRCA1	BRCA1 DNA repair associated	17q21.31	672	NM_007294	Tumor suppressor gene	Genome maintenance	Core DNA Damage Response
BRCA2	BRCA2 DNA repair associated	13q13.1	675	NM_000059	Tumor suppressor gene	Genome maintenance	Core DNA Damage Response
BRD4	bromodomain containing 4	19p13.12	23476	NM_058243	Oncogene	Transcriptional regulation	Transcriptional regulation
BRIP1	BRCA1 interacting protein C-terminal helicase 1	17q23.2	83990	NM_032043	Tumor suppressor gene	Genome maintenance	Core DNA Damage Response
BTG1	BTG anti-proliferation factor 1	12q21.33	694	NM_001731	Tumor suppressor gene	Transcriptional regulation	RNA metabolism
BTG2	BTG anti-proliferation factor 2	1q32.1	7832	NM_006763	---	Cell cycle	Cell cycle
BTK	Bruton tyrosine kinase	Xq22.1	695	NM_000061	Oncogene / Tumor suppressor gene	Immune	Immune
CALR	calreticulin	19p13.13	811	NM_004343	Oncogene	Tumor growth and progression	JAK/STAT
CARD11	caspase recruitment domain family member 11	7p22.2	84433	NM_001324281	Oncogene	Tumor growth and progression	NFKB
CASP8	caspase 8	2q33.1	841	NM_033355	Tumor suppressor gene	Cell death	Apoptosis
CBFB	core-binding factor subunit beta	16q22.1	865	NM_001755	Tumor suppressor gene	Transcriptional regulation	Transcriptional regulation
CBL	Cbl proto-oncogene	11q23.3	867	NM_005188	Oncogene / Tumor suppressor gene	Tumor growth and progression	RTK
CCDC6	coiled-coil domain containing 6	10q21.2	8030	NM_005436	Tumor suppressor gene	---	---
CCND1	cyclin D1	11q13.3	595	NM_053056	Oncogene	Cell cycle	Cell cycle
CCND2	cyclin D2	12p13.32	894	NM_001759	Oncogene	Cell cycle	Cell cycle
CCND3	cyclin D3	6p21.1	896	NM_001760	Oncogene	Cell cycle	Cell cycle
CCNE1	cyclin E1	19q12	898	NM_001238	Oncogene	Cell cycle	Cell cycle
CD22	CD22 molecule	19q13.12	933	NM_001771	---	Immune	Immune
CD274	CD274 molecule	9p24.1	29126	NM_014143	Oncogene / Tumor suppressor gene	Immune	Immune
CD70	CD70 molecule	19p13.3	970	NM_001252	---	Immune	Immune
CD74	CD74 molecule	5q33.1	972	NM_001025159	Oncogene	Immune	Immune
CD79A	CD79a molecule	19q13.2	973	NM_001783	Oncogene	Immune	Immune
CD79B	CD79b molecule	17q23.3	974	NM_000626	Oncogene	Immune	Immune
CDA	cytidine deaminase	1p36.12	978	NM_001785	---	Metabolism	Drug metabolism
CDC73	cell division cycle 73	1q31.2	79577	NM_024529	Tumor suppressor gene	Transcriptional regulation	Transcriptional regulation

Supplementary Table S2. Continued

Official gene symbol	Official gene name	Chromosomal location	Official gene ID	RefSeq transcript ID	Classification	Functional classification	Signaling pathway
CDH1	cadherin 1	16q22.1	999	NM_004360	Tumor suppressor gene	Differentiation	WNT
CDH23	cadherin related 23	10q22.1	64072	NM_022124	---	---	---
CDK12	cyclin-dependent kinase 12	17q12	51755	NM_016507	Tumor suppressor gene	Transcriptional regulation	Transcriptional regulation
CDK4	cyclin-dependent kinase 4	12q14.1	1019	NM_000075	Oncogene	Cell cycle	Cell cycle
CDK6	cyclin-dependent kinase 6	7q21.2	1021	NM_001145306	Oncogene	Cell cycle	Cell cycle
CDK8	cyclin-dependent kinase 8	13q12.13	1024	NM_001260	Oncogene	Transcriptional regulation	Transcriptional regulation
CDKN1A	cyclin-dependent kinase inhibitor 1A	6p21.2	1026	NM_001220778	Oncogene / Tumor suppressor gene	Cell cycle	Cell cycle
CDKN1B	cyclin-dependent kinase inhibitor 1B	12p13.1	1027	NM_004064	Tumor suppressor gene	Cell cycle	Cell cycle
CDKN2A	cyclin-dependent kinase inhibitor 2A	9p21.3	1029	NM_000077	Tumor suppressor gene	Cell cycle	Cell cycle
CDKN2B	cyclin-dependent kinase inhibitor 2B	9p21.3	1030	NM_004936	Tumor suppressor gene	Cell cycle	Cell cycle
CDKN2C	cyclin-dependent kinase inhibitor 2C	1p32.3	1031	NM_001262	Tumor suppressor gene	Cell cycle	Cell cycle
CEBPA	CCAAT enhancer binding protein alpha	19q13.11	1050	NM_004364	Tumor suppressor gene	Transcriptional regulation	Transcriptional regulation
CHCHD7	coiled-coil-helix-coiled-coil-helix domain containing 7	8q12.1	79145	NM_001011671	---	---	---
CHEK1	checkpoint kinase 1	11q24.2	1111	NM_001114122	Tumor suppressor gene	Genome maintenance	TP53
CHEK2	checkpoint kinase 2	22q12.1	11200	NM_007194	Oncogene / Tumor suppressor gene	Genome maintenance	TP53
CIC	capicua transcriptional repressor	19q13.2	23152	NM_015125	Oncogene / Tumor suppressor gene	Tumor growth and progression	MAPK
COL1A1	collagen type I alpha 1 chain	17q21.33	1277	NM_000088	---	---	---
COMT	catechol-O-methyltransferase	22q11.21	1312	NM_000754	---	Metabolism	Drug metabolism
CREBBP	CREB binding protein	16p13.3	1387	NM_004380	Oncogene / Tumor suppressor gene	Epigenetic regulation	Epigenetic modification
CRKL	CRK-like proto-oncogene, adaptor protein	22q11.21	1399	NM_005207	Oncogene	Tumor growth and progression	PI3K/Akt/mTOR
CRLF2	cytokine receptor-like factor 2	Xp22.33 / Yp11.2	64109	NM_022148	Oncogene	Tumor growth and progression	JAK/STAT
CRTC3	CREB regulated transcription coactivator 3	15q26.1	64784	NM_022769	---	Transcriptional regulation	Transcriptional regulation
CSF1R	colony-stimulating factor 1 receptor	5q32	1436	NM_001288705	Oncogene	Immune	Immune
CSF3R	colony-stimulating factor 3 receptor	1p34.3	1441	NM_000760	Oncogene	Tumor growth and progression	JAK/STAT
CTCF	CCCTC-binding factor	16q22.1	10664	NM_006565	Tumor suppressor gene	Epigenetic regulation	Epigenetic modification
CTLA4	cytotoxic T-lymphocyte-associated protein 4	2q33.2	1493	NM_005214	Oncogene	Immune	Immune
CTNNA1	catenin alpha 1	5q31.2	1495	NM_001323982	---	Differentiation	WNT
CTNNB1	catenin beta 1	3p22.1	1499	NM_001904	Oncogene	Differentiation	WNT
CUL3	cullin 3	2q36.2	8452	NM_003590	Tumor suppressor gene	Oxidative stress response	KEAP1/NRF2
CUL4A	cullin 4A	13q34	8451	NM_001008895	---	Protein homeostasis	Protein homeostasis
CXCR4	C-X-C motif chemokine receptor 4	2q22.1	7852	NM_003467	Oncogene	Tumor growth and progression	GPCR
CYLD	CYLD lysine 63 deubiquitinase	16q12.1	1540	NM_015247	Tumor suppressor gene	Protein homeostasis	Protein homeostasis
CYP17A1	cytochrome P450 family 17 subfamily A member 1	10q24.32	1586	NM_000102	---	Metabolism	Metabolic pathway
CYP1A2	cytochrome P450 family 1 subfamily A member 2	15q24.1	1544	NM_000761	---	Metabolism	Drug metabolism
CYP2A6	cytochrome P450 family 2 subfamily A member 6	19q13.2	1548	NM_000762	---	Metabolism	Drug metabolism
CYP2B6	cytochrome P450 family 2 subfamily B member 6	19q13.2	1555	NM_000767	---	Metabolism	Drug metabolism
CYP2C19	cytochrome P450 family 2 subfamily C member 19	10q23.33	1557	NM_000769	---	Metabolism	Drug metabolism
CYP2C9	cytochrome P450 family 2 subfamily C member 9	10q23.33	1559	NM_000771	---	Metabolism	Drug metabolism
CYP2D6	cytochrome P450 family 2 subfamily D member 6	22q13.2	1565	NM_000106	---	Metabolism	Drug metabolism
CYP2E1	cytochrome P450 family 2 subfamily E member 1	10q26.3	1571	NM_000773	---	Metabolism	Drug metabolism
CYP3A4	cytochrome P450 family 3 subfamily A member 4	7q22.1	1576	NM_017460	---	Metabolism	Drug metabolism
CYP3A43	cytochrome P450 family 3 subfamily A member 43	7q22.1	64816	NM_057095	---	Metabolism	Metabolic pathway
CYP3A5	cytochrome P450 family 3 subfamily A member 5	7q22.1	1577	NM_000777	---	Metabolism	Drug metabolism
DAXX	death domain-associated protein	6p21.32	1616	NM_001141969	Oncogene / Tumor suppressor gene	Cell death	Apoptosis
DDIT3	DNA damage inducible transcript 3	12q13.3	1649	NM_004083	Oncogene	Transcriptional regulation	Transcriptional regulation
DDR1	discoidin domain receptor tyrosine kinase 1	6p21.33	780	NM_013993	---	Tumor growth and progression	RTK
DDR2	discoidin domain receptor tyrosine kinase 2	1q23.3	4921	NM_001014796	Oncogene	Tumor growth and progression	RTK
DICER1	dicer 1, ribonuclease III	14q32.13	23405	NM_030621	Tumor suppressor gene	Transcriptional regulation	RNA metabolism
DIS3	DIS3 homolog, exosome endoribonuclease and 3'-5' exoribonuclease	13q21.33	22894	NM_014953	---	Transcriptional regulation	RNA metabolism
DNAAF1	dynein axonemal assembly factor 1	16q24.1	123872	NM_178452	---	---	---
DNMT1	DNA methyltransferase 1	19p13.2	1786	NM_001379	Oncogene	Epigenetic regulation	Epigenetic modification
DNMT3A	DNA methyltransferase 3 alpha	2p23.3	1788	NM_175629	Oncogene / Tumor suppressor gene	Epigenetic regulation	Epigenetic modification
DOT1L	DOT1 like histone lysine methyltransferase	19p13.3	84444	NM_032482	---	Epigenetic regulation	Epigenetic modification
DPYD	dihydropyrimidine dehydrogenase	1p21.3	1806	NM_000110	---	Metabolism	Drug metabolism
EED	embryonic ectoderm development	11q14.2	8726	NM_003797	Tumor suppressor gene	Epigenetic regulation	Epigenetic modification
EGFR	epidermal growth factor receptor	7p11.2	1956	NM_005228	Oncogene	Tumor growth and progression	RTK
EIF3E	eukaryotic translation initiation factor 3 subunit E	8q23.1	3646	NM_001568	Tumor suppressor gene	Transcriptional regulation	RNA metabolism
EML4	EMAP-like 4	2p21	27436	NM_019063	---	---	---
EMSY	EMSY transcriptional repressor, BRCA2 interacting	11q13.5	56946	NM_020193	---	---	---
ENG	endoglin	9q34.11	2022	NM_001114753	---	Tumor growth and progression	TGF-B
ENO1	enolase 1	1p36.23	2023	NM_001428	---	Protein homeostasis	Protein homeostasis
EP300	E1A binding protein p300	22q13.2	2033	NM_001429	Tumor suppressor gene	Epigenetic regulation	Epigenetic modification
EPAS1	endothelial PAS domain protein 1	2p21	2034	NM_001430	Oncogene / Tumor suppressor gene	Tumor growth and progression	PI3K/Akt/mTOR
EPCAM	epithelial cell adhesion molecule	2p21	4072	NM_002354	Tumor suppressor gene	---	---
EPHA3	EPH receptor A3	3p11.1	2042	NM_005233	Tumor suppressor gene	Tumor growth and progression	RTK
EPHB1	EPH receptor B1	3q22.2	2047	NM_004441	---	Tumor growth and progression	RTK
EPHB4	EPH receptor B4	7q22.1	2050	NM_004444	---	Tumor growth and progression	RTK
ERBB2	erb-b2 receptor tyrosine kinase 2	17q12	2064	NM_004448	Oncogene	Tumor growth and progression	RTK
ERBB3	erb-b2 receptor tyrosine kinase 3	12q13.2	2065	NM_001982	Oncogene	Tumor growth and progression	RTK
ERBB4	erb-b2 receptor tyrosine kinase 4	2q34	2066	NM_005235	Oncogene / Tumor suppressor gene	Tumor growth and progression	RTK
ERCC2	ERCC excision repair 2, TFIIH core complex helicase subunit	19q13.32	2068	NM_000400	Tumor suppressor gene	Genome maintenance	Core DNA Damage Response
ERCC4	ERCC excision repair 4, endonuclease catalytic subunit	16p13.12	2072	NM_005236	Tumor suppressor gene	Genome maintenance	Core DNA Damage Response

Supplementary Table S2. Continued

Official gene symbol	Official gene name	Chromosomal location	Official gene ID	RefSeq transcript ID	Classification	Functional classification	Signaling pathway
ERG	ETS transcription factor ERG	21q22.2	2078	NM_001243428	Oncogene	Transcriptional regulation	Transcriptional regulation
ERRFI1	ERBB receptor feedback inhibitor 1	1p36.23	54206	NM_018948	Tumor suppressor gene	Tumor growth and progression	RTK
ESR1	estrogen receptor 1	6q25.1-q25.2	2099	NM_001122742	Oncogene / Tumor suppressor gene	Tumor growth and progression	Nuclear receptor
ETV4	ETS variant transcription factor 4	17q21.31	2118	NM_001079675	Oncogene	Transcriptional regulation	Transcriptional regulation
ETV5	ETS variant transcription factor 5	3q27.2	2119	NM_004454	Oncogene	Transcriptional regulation	Transcriptional regulation
ETV6	ETS variant transcription factor 6	12p13.2	2120	NM_001987	Tumor suppressor gene	Transcriptional regulation	Transcriptional regulation
EWSR1	EWS RNA-binding protein 1	22q12.2	2130	NM_005243	Oncogene	Transcriptional regulation	RNA metabolism
EXT1	exostosin glycosyltransferase 1	8q24.11	2131	NM_000127	Tumor suppressor gene	Metabolism	Metabolic pathway
EXT2	exostosin glycosyltransferase 2	11p11.2	2132	NM_207122	Tumor suppressor gene	Metabolism	Metabolic pathway
EZH2	enhancer of zeste 2 polycomb repressive complex 2 subunit	7q36.1	2146	NM_001203247	Oncogene / Tumor suppressor gene	Epigenetic regulation	Epigenetic modification
EZR	ezrin	6q25.3	7430	NM_003379	---	---	---
TENT5C	terminal nucleotidyltransferase 5C	1p12	54855	NM_017709	Tumor suppressor gene	Transcriptional regulation	RNA metabolism
FANCA	FA complementation group A	16q24.3	2175	NM_000135	Tumor suppressor gene	Genome maintenance	Core DNA Damage Response
FANCC	FA complementation group C	9q22.32	2176	NM_000136	Tumor suppressor gene	Genome maintenance	Core DNA Damage Response
FANCG	FA complementation group G	9p13.3	2189	NM_004629	Tumor suppressor gene	Genome maintenance	DNA damage control
FANCL	FA complementation group L	2p16.1	55120	NM_018062	---	Genome maintenance	Core DNA Damage Response
FAS	Fas cell surface death receptor	10q23.31	355	NM_000043	Tumor suppressor gene	Cell death	Apoptosis
FAT1	FAT atypical cadherin 1	4q35.2	2195	NM_005245	Tumor suppressor gene	Differentiation	WNT
FBXW7	F-box and WD repeat domain containing 7	4q31.3	55294	NM_033632	Tumor suppressor gene	Protein homeostasis	Protein homeostasis
FGF10	fibroblast growth factor 10	5p12	2255	NM_004465	---	Tumor growth and progression	RTK
FGF12	fibroblast growth factor 12	3q28-q29	2257	NM_021032	---	Tumor growth and progression	RTK
FGF14	fibroblast growth factor 14	13q33.1	2259	NM_004115	---	Tumor growth and progression	RTK
FGF19	fibroblast growth factor 19	11q13.3	9965	NM_005117	Oncogene	Tumor growth and progression	RTK
FGF23	fibroblast growth factor 23	12p13.32	8074	NM_020638	---	Tumor growth and progression	RTK
FGF3	fibroblast growth factor 3	11q13.3	2248	NM_005247	Oncogene	Tumor growth and progression	RTK
FGF4	fibroblast growth factor 4	11q13.3	2249	NM_002007	Oncogene	Tumor growth and progression	RTK
FGF6	fibroblast growth factor 6	12p13.32	2251	NM_020996	---	Tumor growth and progression	RTK
FGFR1	fibroblast growth factor receptor 1	8p11.23	2260	NM_023110	Oncogene	Tumor growth and progression	RTK
FGFR2	fibroblast growth factor receptor 2	10q26.13	2263	NM_000141	Oncogene	Tumor growth and progression	RTK
FGFR3	fibroblast growth factor receptor 3	4p16.3	2261	NM_000142	Oncogene	Tumor growth and progression	RTK
FGFR4	fibroblast growth factor receptor 4	5q35.2	2264	NM_213647	Oncogene	Tumor growth and progression	RTK
FH	fumarate hydratase	1q43	2271	NM_000143	Tumor suppressor gene	Metabolism	Metabolic pathway
FLCN	folliculin	17p11.2	201163	NM_144997	Tumor suppressor gene	Tumor growth and progression	PI3K/Akt/mTOR
FLT1	fms-related receptor tyrosine kinase 1	13q12.3	2321	NM_002019	Oncogene	Tumor growth and progression	RTK
FLT3	fms-related receptor tyrosine kinase 3	13q12.2	2322	NM_004119	Oncogene	Tumor growth and progression	RTK
FOXL2	forkhead box L2	3q22.3	668	NM_023067	Oncogene / Tumor suppressor gene	Transcriptional regulation	Transcriptional regulation
FUBP1	far upstream element binding protein 1	1p31.1	8880	NM_003902	Oncogene / Tumor suppressor gene	Transcriptional regulation	Transcriptional regulation
FUS	FUS RNA-binding protein	16p11.2	2521	NM_004960	Tumor suppressor gene	Transcriptional regulation	Transcriptional regulation
G6PD	glucose-6-phosphate dehydrogenase	Xq28	2539	NM_001042351	---	Metabolism	Metabolic pathway
GABRA6	gamma-aminobutyric acid type A receptor subunit alpha6	5q34	2559	NM_000811	---	---	---
GALNT12	polypeptide N-acetylgalactosaminyltransferase 12	9q22.33	79695	NM_024642	---	Metabolism	Metabolic pathway
GATA3	GATA-binding protein 3	10p14	2625	NM_002051	Oncogene / Tumor suppressor gene	Transcriptional regulation	Transcriptional regulation
GATA4	GATA-binding protein 4	8p23.1	2626	NM_002052	---	Transcriptional regulation	Transcriptional regulation
GATA6	GATA-binding protein 6	18q11.2	2627	NM_005257	---	Transcriptional regulation	Transcriptional regulation
GID4	GID complex subunit 4 homolog	17p11.2	79018	NM_024052	---	---	---
GNA11	G protein subunit alpha 11	19p13.3	2767	NM_002067	Oncogene	Tumor growth and progression	GPCR
GNA13	G protein subunit alpha 13	17q24.1	10672	NM_006572	Oncogene	Tumor growth and progression	GPCR
GNAQ	G protein subunit alpha q	9q21.2	2776	NM_002072	Oncogene	Tumor growth and progression	GPCR
GNAS	GNAS complex locus	20q13.32	2778	NM_080425	Oncogene	Tumor growth and progression	GPCR
GRM3	glutamate metabotropic receptor 3	7q21.11-q21.12	2913	NM_000840	Oncogene	Tumor growth and progression	GPCR
GSK3B	glycogen synthase kinase 3 beta	3q13.33	2932	NM_001146156	Oncogene / Tumor suppressor gene	Differentiation	WNT
H3-3A	H3.3 histone A	1q42.12	3020	NM_002107	Oncogene	Epigenetic regulation	Epigenetic modification
HDAC1	histone deacetylase 1	1p35.2-p35.1	3065	NM_004964	Oncogene	Epigenetic regulation	Epigenetic modification
HDAC2	histone deacetylase 2	6q21	3066	NM_001527	---	Differentiation	NOTCH
HEY1	HES-related family bHLH transcription factor with YRPW motif 1	8q21.13	23462	NM_012258	Oncogene / Tumor suppressor gene	Differentiation	NOTCH
HGF	hepatocyte growth factor	7q21.11	3082	NM_000601	Oncogene	Tumor growth and progression	RTK
HMGA2	high-mobility group AT-hook 2	12q14.3	8091	NM_003483	Oncogene	Transcriptional regulation	Transcriptional regulation
HNF1A	HNF1 homeobox A	12q24.31	6927	NM_000545	Oncogene / Tumor suppressor gene	Transcriptional regulation	Transcriptional regulation
HOXB13	homeobox B13	17q21.32	10481	NM_006361	Oncogene / Tumor suppressor gene	Transcriptional regulation	Transcriptional regulation
HRAS	HRas proto-oncogene, GTPase	11p15.5	3265	NM_001130442	Oncogene	Tumor growth and progression	MAPK
HSD3B1	hydroxy-delta-5-steroid dehydrogenase, 3 beta- and steroid delta-isomerase 1	1p12	3283	NM_000862	---	Metabolism	Metabolic pathway
ID3	inhibitor of DNA binding 3, HLH protein	1p36.12	3399	NM_002167	Tumor suppressor gene	Transcriptional regulation	Transcriptional regulation
IDH1	isocitrate dehydrogenase (NADP(+)) 1	2q34	3417	NM_001282387	Oncogene	Epigenetic regulation	Epigenetic modification
IDH2	isocitrate dehydrogenase (NADP(+)) 2	15q26.1	3418	NM_002168	Oncogene	Epigenetic regulation	Epigenetic modification
IGF1R	insulin-like growth factor 1 receptor	15q26.3	3480	NM_000875	Oncogene	Tumor growth and progression	RTK
IGF2	insulin-like growth factor 2	11p15.5	3481	NM_000612	Oncogene	Tumor growth and progression	RTK
IKBKE	inhibitor of nuclear factor kappa B kinase subunit epsilon	1q32.1	9641	NM_014002	Oncogene	Immune	Immune
IKZF1	IKAROS family zinc finger 1	7p12.2	10320	NM_006060	Tumor suppressor gene	Transcriptional regulation	Transcriptional regulation
IL7R	interleukin 7 receptor	5p13.2	3575	NM_002185	Oncogene	Immune	Immune
INPP4B	inositol polyphosphate-4-phosphatase type II B	4q31.21	8821	NM_003866	Tumor suppressor gene	Tumor growth and progression	PI3K/Akt/mTOR
IRF2	interferon regulatory factor 2	4q35.1	3660	NM_002199	---	Immune	Immune
IRF4	interferon regulatory factor 4	6p25.3	3662	NM_002460	Oncogene / Tumor suppressor gene	Immune	Immune

Supplementary Table S2. Continued

Official gene symbol	Official gene name	Chromosomal location	Official gene ID	RefSeq transcript ID	Classification	Functional classification	Signaling pathway
IRS2	insulin receptor substrate 2	13q34	8660	NM_003749	Oncogene	Tumor growth and progression	RTK
JAK1	Janus kinase 1	1p31.3	3716	NM_001321853	Oncogene / Tumor suppressor gene	Tumor growth and progression	JAK/STAT
JAK2	Janus kinase 2	9p24.1	3717	NM_001322194	Oncogene	Tumor growth and progression	JAK/STAT
JAK3	Janus kinase 3	19p13.11	3718	NM_000215	Oncogene	Tumor growth and progression	JAK/STAT
JUN	Jun proto-oncogene, AP-1 transcription factor subunit	1p32.1	3725	NM_002228	Oncogene	Transcriptional regulation	Transcriptional regulation
KDM5A	lysine demethylase 5A	12p13.33	5927	NM_001042603	Oncogene	Epigenetic regulation	Epigenetic modification
KDM5C	lysine demethylase 5C	Xp11.22	8242	NM_004187	Tumor suppressor gene	Epigenetic regulation	Epigenetic modification
KDM6A	lysine demethylase 6A	Xp11.3	7403	NM_021140	Oncogene / Tumor suppressor gene	Epigenetic regulation	Epigenetic modification
KDR	kinase insert domain receptor	4q12	3791	NM_002253	Oncogene	Tumor growth and progression	RTK
KEAP1	Kelch-like ECH-associated protein 1	19p13.2	9817	NM_203500	Tumor suppressor gene	Oxidative stress response	KEAP1/NRF2
KEL	Kell metallo-endopeptidase (Kell blood group)	7q34	3792	NM_000420	---	---	---
KIAA1549	KIAA1549	7q34	57670	NM_001164665	---	---	---
KIF1B	kinesin family member 1B	1p36.22	23095	NM_015074	---	---	---
KIF5B	kinesin family member 5B	10p11.22	3799	NM_004521	---	---	---
KIT	KIT proto-oncogene, receptor tyrosine kinase	4q12	3815	NM_000222	Oncogene	Tumor growth and progression	RTK
KLF4	Kruppel-like factor 4	9q31.2	9314	NM_001314052	Oncogene / Tumor suppressor gene	Transcriptional regulation	Transcriptional regulation
KLHL6	Kelch-like family member 6	3q27.1	89857	NM_130446	---	---	---
KMT2A	lysine methyltransferase 2A	11q23.3	4297	NM_005933	Oncogene / Tumor suppressor gene	Epigenetic regulation	Epigenetic modification
KMT2C	lysine methyltransferase 2C	7q36.1	58508	NM_170606	Tumor suppressor gene	Epigenetic regulation	Epigenetic modification
KMT2D	lysine methyltransferase 2D	12q13.12	8085	NM_003482	Oncogene / Tumor suppressor gene	Epigenetic regulation	Epigenetic modification
KNSTRN	kinetochore-localized astrin (SPAG5)-binding protein	15q15.1	90417	NM_033286	Oncogene	Cell cycle	Cell division
KRAS	KRAS proto-oncogene, GTPase	12p12.1	3845	NM_033360	Oncogene	Tumor growth and progression	MAPK
LMO1	LIM domain only 1	11p15.4	4004	NM_002315	Oncogene	Transcriptional regulation	Transcriptional regulation
LRP5	LDL receptor-related protein 5	11q13.2	4041	NM_002335	Oncogene	Differentiation	WNT
LTK	leukocyte receptor tyrosine kinase	15q15.1	4058	NM_002344	---	---	---
LYN	LYN proto-oncogene, Src family tyrosine kinase	8q12.1	4067	NM_002350	Oncogene	Immune	Immune
LZTR1	leucine zipper-like transcription regulator 1	22q11.21, 22q11.1-q11.2	8216	NM_006767	Tumor suppressor gene	Tumor growth and progression	MAPK
MAF	MAF bZIP transcription factor	16q23.2	4094	NM_001031804	Oncogene / Tumor suppressor gene	Transcriptional regulation	Transcriptional regulation
MAML2	mastermind-like transcriptional coactivator 2	11q21	84441	NM_032427	Oncogene	Differentiation	NOTCH
MAP2K1	mitogen-activated protein kinase kinase 1	15q22.31	5604	NM_002755	Oncogene	Tumor growth and progression	MAPK
MAP2K2	mitogen-activated protein kinase kinase 2	19p13.3	5605	NM_030662	Oncogene	Tumor growth and progression	MAPK
MAP2K4	mitogen-activated protein kinase kinase 4	17p12	6416	NM_003010	Oncogene / Tumor suppressor gene	Tumor growth and progression	MAPK
MAP3K1	mitogen-activated protein kinase kinase kinase 1	5q11.2	4214	NM_005921	Oncogene / Tumor suppressor gene	Tumor growth and progression	MAPK
MAP3K13	mitogen-activated protein kinase kinase kinase 13	3q27.2	9175	NM_004721	Oncogene / Tumor suppressor gene	Tumor growth and progression	MAPK
MAP3K4	mitogen-activated protein kinase kinase kinase 4	6q26	4216	NM_005922	---	Tumor growth and progression	MAPK
MAPK1	mitogen-activated protein kinase 1	22q11.22	5594	NM_002745	Oncogene	Tumor growth and progression	MAPK
MAX	MYC-associated factor X	14q23.3	4149	NM_002382	Oncogene / Tumor suppressor gene	Tumor growth and progression	MYC
MC1R	melanocortin 1 receptor	16q24.3	4157	NM_002386	---	---	---
MCL1	MCL1 apoptosis regulator, BCL2 family member	1q21.2	4170	NM_021960	Oncogene	Cell death	Apoptosis
MDM2	MDM2 proto-oncogene	12q15	4193	NM_002392	Oncogene	Genome maintenance	TP53
MDM4	MDM4 regulator of p53	1q32.1	4194	NM_002393	Oncogene	Genome maintenance	TP53
MED12	mediator complex subunit 12	Xq13.1	9968	NM_005120	Oncogene / Tumor suppressor gene	Transcriptional regulation	Transcriptional regulation
MEF2B	myocyte enhancer factor 2B	19p13.11	100271849	NM_001145785	Oncogene	Transcriptional regulation	Transcriptional regulation
MEN1	menin 1	11q13.1	4221	NM_130803	Tumor suppressor gene	Epigenetic regulation	Epigenetic modification
MERTK	MER proto-oncogene, tyrosine kinase	2q13	10461	NM_006343	---	---	---
MET	MET proto-oncogene, receptor tyrosine kinase	7q31.2	4233	NM_000245	Oncogene	Tumor growth and progression	RTK
MITF	melanocyte-inducing transcription factor	3p13	4286	NM_198159	Oncogene / Tumor suppressor gene	Transcriptional regulation	Transcriptional regulation
MKNK1	MAPK-interacting serine/threonine kinase 1	1p33	8569	NM_003684	---	Tumor growth and progression	MAPK
MKRN1	makorin ring finger protein 1	7q34	23608	NM_013446	---	Protein homeostasis	Protein homeostasis
MLH1	mutL homolog 1	3p22.2	4292	NM_000249	Oncogene / Tumor suppressor gene	Genome maintenance	Core DNA Damage Response
MPL	MPL proto-oncogene, thrombopoietin receptor	1p34.2	4352	NM_005373	Oncogene	Tumor growth and progression	JAK/STAT
MRE11	MRE11 homolog, double-strand break repair nuclease	11q21	4361	NM_005591	---	Genome maintenance	Core DNA Damage Response
MSH2	mutS homolog 2	2p21-p16.3	4436	NM_000251	Tumor suppressor gene	Genome maintenance	Core DNA Damage Response
MSH3	mutS homolog 3	5q14.1	4437	NM_002439	Tumor suppressor gene	Genome maintenance	Core DNA Damage Response
MSH6	mutS homolog 6	2p16.3	2956	NM_000179	Tumor suppressor gene	Genome maintenance	Core DNA Damage Response
MST1R	macrophage-stimulating 1 receptor	3p21.31	4486	NM_002447	Oncogene	Tumor growth and progression	RTK
MTAP	methylthioadenosine phosphorylase	9p21.3	4507	NM_002451	---	Metabolism	Metabolic pathway
MTHFR	methylenetetrahydrofolate reductase	1p36.22	4524	NM_005957	---	Metabolism	Drug metabolism
MTOR	mechanistic target of rapamycin kinase	1p36.22	2475	NM_004958	Oncogene	Tumor growth and progression	PI3K/Akt/mTOR
MTRR	5-methyltetrahydrofolate-homocysteine methyltransferase reductase	5p15.31	4552	NM_024010	---	Metabolism	Metabolic pathway
MUTYH	mutY DNA glycosylase	1p34.1	4595	NM_012222	Tumor suppressor gene	Genome maintenance	DNA damage control
MXI1	MAX interactor 1, dimerization protein	10q25.2	4601	NM_005962	Tumor suppressor gene	Tumor growth and progression	MYC
MYB	MYB proto-oncogene, transcription factor	6q23.3	4602	NM_005375	Oncogene	Transcriptional regulation	Transcriptional regulation
MYC	MYC proto-oncogene, bHLH transcription factor	8q24.21	4609	NM_002467	Oncogene	Tumor growth and progression	MYC
MYCL	MYCL proto-oncogene, bHLH transcription factor	1p34.2	4610	NM_001033081	Oncogene	Tumor growth and progression	MYC
MYCN	MYCN proto-oncogene, bHLH transcription factor	2p24.3	4613	NM_001293228	Oncogene	Tumor growth and progression	MYC
MYD88	MYD88 innate immune signal transduction adaptor	3p22.2	4615	NM_002468	Oncogene	Tumor growth and progression	NFKB
NAT2	N-acetyltransferase 2	8p22	10	NM_000015	---	Metabolism	Drug metabolism
NBN	nibrin	8q21.3	4683	NM_002485	Tumor suppressor gene	Genome maintenance	Core DNA Damage Response
NCF2	neutrophil cytosolic factor 2	1q25.3	4688	NM_000433	---	---	---
NCOA2	nuclear receptor coactivator 2	8q13.3	10499	NM_001321703	Oncogene	Epigenetic regulation	Epigenetic modification
NCOA3	nuclear receptor coactivator 3	20q13.12	8202	NM_181659	Oncogene	Epigenetic regulation	Epigenetic modification

Supplementary Table S2. Continued

Official gene symbol	Official gene name	Chromosomal location	Official gene ID	RefSeq transcript ID	Classification	Functional classification	Signaling pathway
NCOA4	nuclear receptor coactivator 4	10q11.22	8031	NM_001145262	Tumor suppressor gene	Transcriptional regulation	Transcriptional regulation
NCOR1	nuclear receptor corepressor 1	17p12-p11.2	9611	NM_006311	Tumor suppressor gene	Epigenetic regulation	Epigenetic modification
NF1	neurofibromin 1	17q11.2	4763	NM_001042492	Tumor suppressor gene	Tumor growth and progression	MAPK
NF2	neurofibromin 2	22q12.2	4771	NM_000268	Tumor suppressor gene	Tumor growth and progression	Hippo
NFE2L2	nuclear factor, erythroid 2-like 2	2q31.2	4780	NM_006164	Oncogene / Tumor suppressor gene	Oxidative stress response	KEAP1/NRF2
NFIB	nuclear factor I B	9p23-p22.3	4781	NM_005596	---	Transcriptional regulation	Transcriptional regulation
NFKBIA	NFKB inhibitor alpha	14q13.2	4792	NM_020529	Tumor suppressor gene	Tumor growth and progression	NFKB
NKX2-1	NK2 homeobox 1	14q13.3	7080	NM_003317	Oncogene / Tumor suppressor gene	Transcriptional regulation	Transcriptional regulation
NOTCH1	notch receptor 1	9q34.3	4851	NM_017617	Oncogene / Tumor suppressor gene	Differentiation	NOTCH
NOTCH2	notch receptor 2	1p12	4853	NM_024408	Oncogene / Tumor suppressor gene	Differentiation	NOTCH
NOTCH3	notch receptor 3	19p13.12	4854	NM_000435	Oncogene / Tumor suppressor gene	Differentiation	NOTCH
NPM1	nucleophosmin 1	5q35.1	4869	NM_002520	Oncogene / Tumor suppressor gene	Epigenetic regulation	Epigenetic modification
NRAS	NRAS proto-oncogene, GTPase	1p13.2	4893	NM_002524	Oncogene	Tumor growth and progression	MAPK
NRG1	neuregulin 1	8p12	3084	NM_013964	Tumor suppressor gene	Tumor growth and progression	RTK
NT5C2	5'-nucleotidase, cytosolic II	10q24.32-q24.33	22978	NM_001134373	Oncogene	Metabolism	Metabolic pathway
NTRK1	neurotrophic receptor tyrosine kinase 1	1q23.1	4914	NM_002529	Oncogene / Tumor suppressor gene	Tumor growth and progression	RTK
NTRK2	neurotrophic receptor tyrosine kinase 2	9q21.33	4915	NM_001018064	Oncogene	Tumor growth and progression	RTK
NTRK3	neurotrophic receptor tyrosine kinase 3	15q25.3	4916	NM_001012338	Oncogene	Tumor growth and progression	RTK
NUTM1	NUT midline carcinoma family member 1	15q14	256646	NM_001284292	Oncogene	---	---
P2RY8	P2Y receptor family member 8	Xp22.33 / Yp11.2	286530	NM_178129	Oncogene / Tumor suppressor gene	Tumor growth and progression	GPCR
PALB2	partner and localizer of BRCA2	16p12.2	79728	NM_024675	Tumor suppressor gene	Genome maintenance	Core DNA Damage Response
PRKN	parkin RBR E3 ubiquitin protein ligase	6q26	5071	NM_004562	Tumor suppressor gene	Protein homeostasis	Protein homeostasis
PARP1	poly(ADP-ribose) polymerase 1	1q42.12	142	NM_001618	Tumor suppressor gene	Genome maintenance	Core DNA Damage Response
PARP2	poly(ADP-ribose) polymerase 2	14q11.2	10038	NM_005484	---	Genome maintenance	DNA damage control
PARP3	poly(ADP-ribose) polymerase family member 3	3p21.2	10039	NM_001003931	---	Genome maintenance	DNA damage control
PAX5	paired box 5	9p13.2	5079	NM_016734	Oncogene / Tumor suppressor gene	Transcriptional regulation	Transcriptional regulation
PBRM1	polybromo 1	3p21.1	55193	NM_018313	Tumor suppressor gene	Epigenetic regulation	Epigenetic modification
PDCD1	programmed cell death 1	2q37.3	5133	NM_005018	Oncogene	Immune	Immune
PDCD1LG2	programmed cell death 1 ligand 2	9p24.1	80380	NM_025239	Oncogene	Immune	Immune
PDE11A	phosphodiesterase 11A	2q31.2	50940	NM_016953	---	Metabolism	Metabolic pathway
PDGFB	platelet-derived growth factor subunit B	22q13.1	5155	NM_002608	Oncogene	Metabolism	Metabolic pathway
PDGFRA	platelet-derived growth factor receptor alpha	4q12	5156	NM_006206	Oncogene	Tumor growth and progression	RTK
PDGFRB	platelet-derived growth factor receptor beta	5q32	5159	NM_002609	Oncogene	Tumor growth and progression	RTK
PDK1	pyruvate dehydrogenase kinase 1	2q31.1	5163	NM_002610	Oncogene	Metabolism	Metabolic pathway
PHOX2B	paired-like homeobox 2B	4p13	8929	NM_003924	Tumor suppressor gene	Transcriptional regulation	Transcriptional regulation
PIK3C2B	phosphatidylinositol-4-phosphate 3-kinase catalytic subunit type 2 beta	1q32.1	5287	NM_002646	---	Tumor growth and progression	PI3K/Akt/mTOR
PIK3C2G	phosphatidylinositol-4-phosphate 3-kinase catalytic subunit type 2 gamma	12p12.3	5288	NM_004570	---	Tumor growth and progression	PI3K/Akt/mTOR
PIK3CA	phosphatidylinositol-4,5-bisphosphate 3-kinase catalytic subunit alpha	3q26.32	5290	NM_006218	Oncogene	Tumor growth and progression	PI3K/Akt/mTOR
PIK3CB	phosphatidylinositol-4,5-bisphosphate 3-kinase catalytic subunit beta	3q22.3	5291	NM_006219	Oncogene	Tumor growth and progression	PI3K/Akt/mTOR
PIK3CG	phosphatidylinositol-4,5-bisphosphate 3-kinase catalytic subunit gamma	7q22.3	5294	NM_002649	Oncogene	Tumor growth and progression	PI3K/Akt/mTOR
PIK3R1	phosphoinositide-3-kinase regulatory subunit 1	5q13.1	5295	NM_181523	Tumor suppressor gene	Tumor growth and progression	PI3K/Akt/mTOR
PIK3R2	phosphoinositide-3-kinase regulatory subunit 2	19p13.11	5296	NM_005027	Oncogene / Tumor suppressor gene	Tumor growth and progression	PI3K/Akt/mTOR
PIM1	Pim-1 proto-oncogene, serine/threonine kinase	6p21.2	5292	NM_001243186	Oncogene	Tumor growth and progression	JAK/STAT
PLAG1	PLAG1 zinc finger	8q12.1	5324	NM_002655	Oncogene	Transcriptional regulation	Transcriptional regulation
PMS1	PMS1 homolog 1, mismatch repair system component	2q32.2	5378	NM_001321047	Tumor suppressor gene	Genome maintenance	Core DNA Damage Response
PMS2	PMS1 homolog 2, mismatch repair system component	7p22.1	5395	NM_000535	Tumor suppressor gene	Genome maintenance	Core DNA Damage Response
POLD1	DNA polymerase delta 1, catalytic subunit	19q13.33	5424	NM_001256849	Tumor suppressor gene	Genome maintenance	DNA damage control
POLE	DNA polymerase epsilon, catalytic subunit	12q24.33	5426	NM_006231	Tumor suppressor gene	Genome maintenance	Core DNA Damage Response
POLH	DNA polymerase eta	6p21.1	5429	NM_006502	---	Genome maintenance	DNA damage control
PPARG	peroxisome proliferator activated receptor gamma	3p25.2	5468	NM_015869	Oncogene / Tumor suppressor gene	Tumor growth and progression	Nuclear receptor
PPP2R1A	protein phosphatase 2 scaffold subunit alpha	19q13.41	5518	NM_014225	Oncogene / Tumor suppressor gene	Tumor growth and progression	PI3K/Akt/mTOR
PPP2R2A	protein phosphatase 2 regulatory subunit alpha	8p21.2	5520	NM_002717	---	Tumor growth and progression	PI3K/Akt/mTOR
PPP6C	protein phosphatase 6 catalytic subunit	9q33.3	5537	NM_002721	Tumor suppressor gene	Epigenetic regulation	Epigenetic modification
PRDM1	PR/SET domain 1	6q21	639	NM_001198	Tumor suppressor gene	Transcriptional regulation	Transcriptional regulation
PRKAR1A	protein kinase cAMP-dependent type I regulatory subunit alpha	17q24.2	5573	NM_212471	Oncogene / Tumor suppressor gene	Tumor growth and progression	PI3K/Akt/mTOR
PRKCI	protein kinase C iota	3q26.2	5584	NM_002740	Oncogene	Differentiation	Hedgehog
PTCH1	patched 1	9q22.32	5727	NM_000264	Tumor suppressor gene	Differentiation	Hedgehog
PTEN	phosphatase and tensin homolog	10q23.31	5728	NM_000314	Tumor suppressor gene	Tumor growth and progression	PI3K/Akt/mTOR
PTPN11	protein tyrosine phosphatase non-receptor type 11	12q24.13	5781	NM_002834	Oncogene	Tumor growth and progression	MAPK
PTPRK	protein tyrosine phosphatase receptor type K	6q22.33	5796	NM_001291984	Tumor suppressor gene	Tumor growth and progression	RTK
PTPRO	protein tyrosine phosphatase receptor type O	12p12.3, 12p13-p12	5800	NM_030667	---	Tumor growth and progression	RTK
PTPRT	protein tyrosine phosphatase receptor type T	20q12-q13.11	11122	NM_007050	Tumor suppressor gene	Tumor growth and progression	RTK
QKI	QKI, KH domain containing RNA binding	6q26	9444	NM_006775	Oncogene / Tumor suppressor gene	Transcriptional regulation	RNA metabolism
RAC1	Rac family small GTPase 1	7p22.1	5879	NM_006908	Oncogene	Tumor growth and progression	MAPK
RAC2	Rac family small GTPase 2	22q13.1	5880	NM_002872	Oncogene	Tumor growth and progression	MAPK
RAD21	RAD21 cohesin complex component	8q24.11	5885	NM_006265	Oncogene / Tumor suppressor gene	Cell cycle	Cell division
RAD50	RAD50 double-strand break repair protein	5q31.1	10111	NM_005732	Tumor suppressor gene	Genome maintenance	Core DNA Damage Response
RAD51	RAD51 recombinase	15q15.1	5888	NM_002875	Tumor suppressor gene	Genome maintenance	Core DNA Damage Response
RAD51B	RAD51 paralog B	14q24.1	5890	NM_133509	Tumor suppressor gene	Genome maintenance	DNA damage control
RAD51C	RAD51 paralog C	17q22	5889	NM_058216	Tumor suppressor gene	Genome maintenance	DNA damage control
RAD51D	RAD51 paralog D	17q12	5892	NM_002878	Tumor suppressor gene	Genome maintenance	DNA damage control
RAD52	RAD52 homolog, DNA repair protein	12p13.33	5893	NM_001297419	---	Genome maintenance	Core DNA Damage Response

Supplementary Table S2. Continued

Official gene symbol	Official gene name	Chromosomal location	Official gene ID	RefSeq transcript ID	Classification	Functional classification	Signaling pathway
RAD54L	RAD54-like	1p34.1	8438	NM_003579	---	Genome maintenance	DNA damage control
RAF1	Raf-1 proto-oncogene, serine/threonine kinase	3p25.2	5894	NM_002880	Oncogene	Tumor growth and progression	MAPK
RARA	retinoic acid receptor alpha	17q21.2	5914	NM_001145301	Oncogene	Tumor growth and progression	Nuclear receptor
RB1	RB transcriptional corepressor 1	13q14.2	5925	NM_000321	Tumor suppressor gene	Cell cycle	Cell cycle
RBBP6	RB-binding protein 6, ubiquitin ligase	16p12.1	5930	NM_006910	---	Protein homeostasis	Protein homeostasis
RBM10	RNA-binding motif protein 10	Xp11.3	8241	NM_005676	Tumor suppressor gene	Transcriptional regulation	RNA metabolism
RECQL4	RecQ-like helicase 4	8q24.3	9401	NM_004260	Oncogene / Tumor suppressor gene	Genome maintenance	DNA damage control
REL	REL proto-oncogene, NF-kB subunit	2p16.1	5966	NM_002908	Oncogene	Tumor growth and progression	NFKB
RET	ret proto-oncogene	10q11.21	5979	NM_020975	Oncogene	Tumor growth and progression	RTK
RHEB	Ras homolog, mTORC1 binding	7q36.1	6009	NM_005614	Oncogene	Tumor growth and progression	PI3K/Akt/mTOR
RHOA	ras homolog family member A	3p21.31	387	NM_001313941	Oncogene / Tumor suppressor gene	Tumor growth and progression	MAPK
RICTOR	RPTOR-independent companion of MTOR complex 2	5p13.1	253260	NM_152756	Oncogene	Tumor growth and progression	PI3K/Akt/mTOR
RIT1	Ras-like without CAAX 1	1q22	6016	NM_006912	Oncogene	Tumor growth and progression	MAPK
RNF43	ring finger protein 43	17q22	54894	NM_017763	Tumor suppressor gene	Differentiation	WNT
ROS1	ROS proto-oncogene 1, receptor tyrosine kinase	6q22.1	6098	NM_002944	Oncogene	Tumor growth and progression	RTK
RPTOR	regulatory-associated protein of MTOR complex 1	17q25.3	57521	NM_020761	Oncogene	Tumor growth and progression	PI3K/Akt/mTOR
RRAS2	RAS-related 2	11p15.2	22800	NM_012250	Oncogene	Tumor growth and progression	MAPK
RSPO2	R-spondin 2	8q23.1	340419	NM_178565	Tumor suppressor gene	Differentiation	WNT
RSPO3	R-spondin 3	6q22.33	84870	NM_032784	Oncogene	Differentiation	WNT
RUNDC1	RUN domain containing 1	17q21.31	146923	NM_173079	---	---	---
RUNX1	RUNX family transcription factor 1	21q22.12	861	NM_001001890	Oncogene / Tumor suppressor gene	Transcriptional regulation	Transcriptional regulation
S1PR3	sphingosine-1-phosphate receptor 3	9q22.1	1903	NM_005226	---	Tumor growth and progression	GPCR
SALL4	spalt-like transcription factor 4	20q13.2	57167	NM_020436	Oncogene	Transcriptional regulation	Transcriptional regulation
SAMD9	sterile alpha motif domain containing 9	7q21.2	54809	NM_017654	---	---	---
SDC4	syndecan 4	20q13.12	6385	NM_002999	---	---	---
SDHA	succinate dehydrogenase complex flavoprotein subunit A	5p15.33	6389	NM_004168	Tumor suppressor gene	Metabolism	Metabolic pathway
SDHAF2	succinate dehydrogenase complex assembly factor 2	11q12.2	54949	NM_017841	Tumor suppressor gene	Metabolism	Metabolic pathway
SDHB	succinate dehydrogenase complex iron sulfur subunit B	1p36.13	6390	NM_003000	Tumor suppressor gene	Metabolism	Metabolic pathway
SDHC	succinate dehydrogenase complex subunit C	1q23.3	6391	NM_003001	Tumor suppressor gene	Metabolism	Metabolic pathway
SDHD	succinate dehydrogenase complex subunit D	11q23.1	6392	NM_003002	Tumor suppressor gene	Metabolism	Metabolic pathway
SETBP1	SET-binding protein 1	18q12.3	26040	NM_015559	Oncogene	Epigenetic regulation	Epigenetic modification
SETD2	SET domain containing 2, histone lysine methyltransferase	3p21.31	29072	NM_014159	Tumor suppressor gene	Epigenetic regulation	Epigenetic modification
SF3B1	splicing factor 3b subunit 1	2q33.1	23451	NM_012433	Oncogene	Transcriptional regulation	RNA metabolism
SGK1	serum/glucocorticoid regulated kinase 1	6q23.2	6446	NM_005627	Oncogene	Tumor growth and progression	PI3K/Akt/mTOR
SH2D1A	SH2 domain containing 1A	Xq25	4068	NM_002351	Tumor suppressor gene	Immune	Immune
SHOC2	SHOC2 leucine-rich repeat scaffold protein	10q25.2	8036	NM_001324337	Oncogene	Tumor growth and progression	MAPK
SKP2	S-phase kinase-associated protein 2	5p13.2	6502	NM_005983	Oncogene	Cell cycle	Cell cycle
SLC22A18	solute carrier family 22 member 18	11p15.4	5002	NM_183233	---	Metabolism	Metabolic pathway
SLC34A2	solute carrier family 34 member 2	4p15.2	10568	NM_006424	Tumor suppressor gene	---	---
SLX4	SLX4 structure-specific endonuclease subunit	16p13.3	84464	NM_032444	Tumor suppressor gene	Genome maintenance	DNA damage control
SMAD2	SMAD family member 2	18q21.1	4087	NM_001003652	Tumor suppressor gene	Tumor growth and progression	TGF-B
SMAD4	SMAD family member 4	18q21.2	4089	NM_005359	Tumor suppressor gene	Tumor growth and progression	TGF-B
SMARCA4	SWI/SNF related, matrix-associated, actin-dependent regulator of chromatin, subfamily a, member 4	19p13.2	6597	NM_001128844	Tumor suppressor gene	Epigenetic regulation	Epigenetic modification
SMARCB1	SWI/SNF-related, matrix-associated, actin-dependent regulator of chromatin, subfamily b, member 1	22q11.23, 22q11	6598	NM_003073	Tumor suppressor gene	Epigenetic regulation	Epigenetic modification
SMO	smoothened, frizzled class receptor	7q32.1	6608	NM_005631	Oncogene	Differentiation	Hedgehog
SNCAIP	synuclein alpha-interacting protein	5q23.2	9627	NM_005460	---	---	---
SOCS1	suppressor of cytokine signaling 1	16p13.13	8651	NM_003745	Tumor suppressor gene	Tumor growth and progression	JAK/STAT
SOS1	SOS Ras/Rac guanine nucleotide exchange factor 1	2p22.1	6654	NM_005633	Oncogene	Tumor growth and progression	MAPK
SOX2	SRY-box transcription factor 2	3q26.33	6657	NM_003106	Oncogene	Transcriptional regulation	Transcriptional regulation
SOX9	SRY-box transcription factor 9	17q24.3	6662	NM_000346	Oncogene / Tumor suppressor gene	Differentiation	WNT
SPEN	spen family transcriptional repressor	1p36.21-p36.13	23013	NM_015001	Tumor suppressor gene	Transcriptional regulation	Transcriptional regulation
SPINK1	serine peptidase inhibitor Kazal type 1	5q32	6690	NM_003122	---	---	---
SPOP	speckle type BTB/POZ protein	17q21.33	8405	NM_001007226	Oncogene / Tumor suppressor gene	Protein homeostasis	Protein homeostasis
SRC	SRC proto-oncogene, non-receptor tyrosine kinase	20q11.23	6714	NM_005417	Oncogene	Tumor growth and progression	MAPK
SS18	SS18 subunit of BAF chromatin remodeling complex	18q11.2	6760	NM_001007559	Oncogene	Transcriptional regulation	Transcriptional regulation
SSX1	SSX family member 1	Xp11.23	6756	NM_001278691	Oncogene	Transcriptional regulation	Transcriptional regulation
STAG2	stromal antigen 2	Xq25	10735	NM_001042751	Tumor suppressor gene	Cell cycle	Cell division
STAT3	signal transducer and activator of transcription 3	17q21.2	6774	NM_139276	Oncogene	Tumor growth and progression	JAK/STAT
STK11	serine/threonine kinase 11	19p13.3	6794	NM_000455	Tumor suppressor gene	Tumor growth and progression	PI3K/Akt/mTOR
STRN	striatin	2p22.2	6801	NM_003162	---	---	---
SUFU	SUFU negative regulator of hedgehog signaling	10q24.32	51684	NM_016169	Tumor suppressor gene	Differentiation	Hedgehog
SYK	spleen-associated tyrosine kinase	9q22.2	6850	NM_003177	Oncogene	Immune	Immune
TACC3	transforming acidic coiled-coil containing protein 3	4p16.3	10460	NM_006342	---	Transcriptional regulation	RNA metabolism
TBX3	T-box transcription factor 3	12q24.21	6926	NM_016569	Oncogene / Tumor suppressor gene	Transcriptional regulation	Transcriptional regulation
TCF7L2	transcription factor 7-like 2	10q25.2-q25.3	6934	NM_001146274	Oncogene / Tumor suppressor gene	Differentiation	WNT
TEK	TEK receptor tyrosine kinase	9p21.2	7010	NM_000459	---	Tumor growth and progression	RTK
TERC	telomerase RNA component	3q26.2	7012	NR_001566	---	Genome maintenance	DNA damage control
TERT	telomerase reverse transcriptase	5p15.33	7015	NM_198253	Oncogene / Tumor suppressor gene	Genome maintenance	DNA damage control
TET2	tet methylcytosine dioxygenase 2	4q24	54790	NM_001127208	Tumor suppressor gene	Epigenetic regulation	Epigenetic modification
TGFBR1	transforming growth factor beta receptor 1	9q22.33	7046	NM_004612	Tumor suppressor gene	Tumor growth and progression	TGF-B
TGFBR2	transforming growth factor beta receptor 2	3p24.1	7048	NM_003242	Tumor suppressor gene	Tumor growth and progression	TGF-B
TIPARP	TCDD-inducible poly(ADP-ribose) polymerase	3q25.31	25976	NM_001184717	---	Metabolism	Metabolic pathway

Supplementary Table S2. Continued

Official gene symbol	Official gene name	Chromosomal location	Official gene ID	RefSeq transcript ID	Classification	Functional classification	Signaling pathway
TMEM127	transmembrane protein 127	2q11.2	55654	NM_017849	Tumor suppressor gene	Tumor growth and progression	PI3K/Akt/mTOR
TMPRSS2	transmembrane serine protease 2	21q22.3	7113	NM_005656	---	Transcriptional regulation	Transcriptional regulation
TNFAIP3	TNF alpha-induced protein 3	6q23.3	7128	NM_001270508	Tumor suppressor gene	Tumor growth and progression	NFKB
TNFRSF14	TNF receptor superfamily member 14	1p36.32	8764	NM_003820	Tumor suppressor gene	Immune	Immune
TNK2	tyrosine kinase non receptor 2	3q29	10188	NM_005781	---	Tumor growth and progression	RTK
TP53	tumor protein p53	17p13.1	7157	NM_000546	Oncogene / Tumor suppressor gene	Genome maintenance	TP53
TP63	tumor protein p63	3q28	8626	NM_003722	Oncogene / Tumor suppressor gene	Transcriptional regulation	Transcriptional regulation
TPM3	tropomyosin 3	1q21.3	7170	NM_152263	Tumor suppressor gene	---	---
TPMT	thiopurine S-methyltransferase	6p22.3	7172	NM_000367	---	Metabolism	Drug metabolism
TRAF7	TNF receptor-associated factor 7	16p13.3	84231	NM_032271	Tumor suppressor gene	Protein homeostasis	Protein homeostasis
TSC1	TSC complex subunit 1	9q34.13	7248	NM_000368	Tumor suppressor gene	Tumor growth and progression	PI3K/Akt/mTOR
TSC2	TSC complex subunit 2	16p13.3	7249	NM_000548	Tumor suppressor gene	Tumor growth and progression	PI3K/Akt/mTOR
TSHR	thyroid-stimulating hormone receptor	14q31.1	7253	NM_000369	Oncogene	Tumor growth and progression	GPCR
TYRO3	TYRO3 protein tyrosine kinase	15q15.1	7301	NM_006293	---	Tumor growth and progression	RTK
U2AF1	U2 small nuclear RNA auxiliary factor 1	21q22.3	7307	NM_006758	Oncogene	Transcriptional regulation	RNA metabolism
UBE2T	ubiquitin-conjugating enzyme E2 T	1q32.1	29089	NM_014176	---	Genome maintenance	Core DNA Damage Response
UGT1A1	UDP glucuronosyltransferase family 1 member A1	2q37.1	54658	NM_000463	---	Metabolism	Drug metabolism
VEGFA	vascular endothelial growth factor A	6p21.1	7422	NM_001171623	Oncogene	Tumor growth and progression	RTK
VHL	von Hippel-Lindau tumor suppressor	3p25.3	7428	NM_000551	Tumor suppressor gene	Tumor growth and progression	PI3K/Akt/mTOR
VTI1A	vesicle transport through interaction with t-SNAREs 1A	10q25.2	143187	NM_145206	---	---	---
NSD2	nuclear receptor binding SET domain protein 2	4p16.3	7468	NM_133330	Oncogene	Epigenetic regulation	Epigenetic modification
NSD3	nuclear receptor binding SET domain protein 3	8p11.23	54904	NM_023034	Oncogene	Epigenetic regulation	Epigenetic modification
WT1	WT1 transcription factor	11p13	7490	NM_024426	Oncogene / Tumor suppressor gene	Transcriptional regulation	Transcriptional regulation
XPA	XPA, DNA damage recognition and repair factor	9q22.33	7507	NM_000380	Tumor suppressor gene	Genome maintenance	Core DNA Damage Response
XPO1	exportin 1	2p15	7514	NM_003400	Oncogene	Transcriptional regulation	RNA metabolism
XRCC2	X-ray repair cross complementing 2	7q36.1	7516	NM_005431	Tumor suppressor gene	Genome maintenance	Core DNA Damage Response
ZNF217	zinc finger protein 217	20q13.2	7764	NM_006526	Oncogene	Transcriptional regulation	Transcriptional regulation
ZNF703	zinc finger protein 703	8p11.23	80139	NM_025069	---	Transcriptional regulation	Transcriptional regulation
