# Supplementary material for: JCGA: the Japanese version of the Cancer Genome Atlas and its contribution to the interpretation of gene alterations detected in clinical cancer genome sequencing
Source: Hum Genome Var. 2021 Sep 30;8:38. doi: 10.1038/s41439-021-00170-w (PMC8481308; doi:10.1038/s41439-021-00170-w)
Supplement: Supplementary file 4 — Supplementary Table S3. List of functional categories and signaling pathways [file 41439_2021_170_MOESM4_ESM.pdf]

Supplementary Table S3. List of functional categories and signaling pathways

Functional category	Signaling pathway	No. of genes	Official gene symbol
Cell cycle	Cell cycle	14	*BTG2, CCND1, CCND2, CCND3, CCNE1, CDK4, CDK6, CDKN1A, CDKN1B, CDKN2A, CDKN2B, CDKN2C, RB1, SKP2*
	Cell division	5	*AURKA, AURKB, KNSTRN, RAD21, STAG2*
Cell death	Apoptosis	9	*BAX, BCL2, BCL2L1, BCL2L11, BCL2L2, CASP8, DAXX, FAS, MCL1*
Tumor growth and progression	GPCR	9	*CXCR4, GNA11, GNA13, GNAQ, GNAS, GRM3, P2RY8, S1PR3, TSHR*
	Hippo	1	*NF2*
	JAK/STAT	10	*CALR, CRLF2, CSF3R, JAK1, JAK2, JAK3, MPL, PIM1, SOCS1, STAT3*
	MAPK	26	*ARAF, BRAF, CIC, HRAS, KRAS, LZTR1, MAP2K1, MAP2K2, MAP2K4, MAP3K1, MAP3K13, MAP3K4, MAPK1, MKNK1, NF1, NRAS, PTPN11, RAC1, RAC2, RAF1, RHOA, RIT1, RRAS2, SHOC2, SOS1, SRC*
	MYC	5	*MAX, MXI1, MYC, MYCL, MYCN*
	NFKB	6	*BCL10, CARD11, MYD88, NFKBIA, REL, TNFAIP3*
	Nuclear receptor	4	*AR, ESR1, PPARG, RARA*
	PI3K/Akt/mTOR	28	*AKT1, AKT2, AKT3, CRKL, EPAS1, FLCN, INPP4B, MTOR, PIK3C2B, PIK3C2G, PIK3CA, PIK3CB, PIK3CG, PIK3R1, PIK3R2, PPP2R1A, PPP2R2A, PRKAR1A, PTEN, RHEB, RICTOR, RPTOR, SGK1, STK11, TMEM127, TSC1, TSC2, VHL*
	RTK	52	*ABL1, ABL2, ALK, AXL, CBL, DDR1, DDR2, EGFR, EPHA3, EPHB1, EPHB4, ERBB2, ERBB3, ERBB4, ERRFI1, FGF10, FGF12, FGF14, FGF19, FGF23, FGF3, FGF4, FGF6, FGFR1, FGFR2, FGFR3, FGFR4, FLT1, FLT3, HGF, IGF1R, IGF2, IRS2, KDR, KIT, MET, MST1R, NRG1, NTRK1, NTRK2, NTRK3, PDGFRA, PDGFRB, PTPRK, PTPRO, PTPRT, RET, ROS1, TEK, TNK2, TYRO3, VEGFA*
	TGF-B	7	*ACVR1B, BMPR1A, ENG, SMAD2, SMAD4, TGFBR1, TGFBR2*
Differentiation	Hedgehog	4	*PRKCI, PTCH1, SMO, SUFU*
	NOTCH	6	*HDAC2, HEY1, MAML2, NOTCH1, NOTCH2, NOTCH3*
	WNT	14	*AMER1, APC, AXIN1, CDH1, CTNNA1, CTNNB1, FAT1, GSK3B, LRP5, RNF43, RSPO2, RSPO3, SOX9, TCF7L2*
Genome maintenance	Core DNA Damage Response	27	*BARD1, BLM, BRCA1, BRCA2, BRIP1, ERCC2, ERCC4, FANCA, FANCC, FANCL, MLH1, MRE11, MSH2, MSH3, MSH6, NBN, PALB2, PARP1, PMS1, PMS2, POLE, RAD50, RAD51, RAD52, UBE2T, XPA, XRCC2*
	DNA damage control	14	*FANCG, MUTYH, PARP2, PARP3, POLD1, POLH, RAD51B, RAD51C, RAD51D, RAD54L, RECQL4, SLX4, TERC, TERT*
	TP53	7	*ATM, ATR, CHEK1, CHEK2, MDM2, MDM4, TP53*
Immune	Immune	22	*ALOX12B, B2M, BCL6, BTK, CD22, CD274, CD70, CD74, CD79A, CD79B, CSF1R, CTLA4, IKBKE, IL7R, IRF2, IRF4, LYN, PDCD1, PDCD1LG2, SH2D1A, SYK, TNFRSF14*
Metabolism	Drug metabolism	19	*ABCB1, ABCG2, ALDH2, CDA, COMT, CYP1A2, CYP2A6, CYP2B6, CYP2C19, CYP2C9, CYP2D6, CYP2E1, CYP3A4, CYP3A5, DPYD, MTHFR, NAT2, TPMT, UGT1A1*
	Metabolic pathway	22	*ADH1B, CYP17A1, CYP3A43, EXT1, EXT2, FH, G6PD, GALNT12, HSD3B1, MTAP, MTRR, NT5C2, PDE11A, PDGFB, PDK1, SDHA, SDHAF2, SDHB, SDHC, SDHD, SLC22A18, TIPARP*
Oxidative stress response	KEAP1/NRF2	3	*CUL3, KEAP1, NFE2L2*
Protein homeostasis	Protein homeostasis	10	*BAP1, CUL4A, CYLD, ENO1, FBXW7, MKRN1, PRKN, RBBP6, SPOP, TRAF7*
Epigenetic regulation	Epigenetic modification	39	*ARID1A, ARID1B, ARID2, ASXL1, ATRX, BCOR, BCORL1, CREBBP, CTCF, DNMT1, DNMT3A, DOT1L, EED, EP300, EZH2, H3-3A, HDAC1, IDH1, IDH2, KDM5A, KDM5C, KDM6A, KMT2A, KMT2C, KMT2D, MEN1, NCOA2, NCOA3, NCOR1, NPM1, PBRM1, PPP6C, SETBP1, SETD2, SMARCA4, SMARCB1, TET2, NSD2, NSD3*
Transcriptional regulation	RNA metabolism	12	*BTG1, DICER1, DIS3, EIF3E, EWSR1, TENT5C, QKI, RBM10, SF3B1, TACC3, U2AF1, XPO1*
	Transcriptional regulation	51	*ATF1, BRD4, CBFB, CDC73, CDK8, CDK12, CEBPA, CRTC3, DDIT3, ERG, ETV4, ETV5, ETV6, FOXL2, FUBP1, FUS, GATA3, GATA4, GATA6, HMGA2, HNF1A, HOXB13, ID3, IKZF1, JUN, KLF4, LMO1, MAF, MED12, MEF2B, MITF, MYB, NCOA4, NFIB, NKX2-1, PAX5, PHOX2B, PLAG1, PRDM1, RUNX1, SALL4, SOX2, SPEN, SS18, SSX1, TBX3, TMPRSS2, TP63, WT1, ZNF217, ZNF703*
Not classified	---	34	*ACTN4, AIP, ARFRP1, BCR, CCDC6, CDH23, CHCHD7, COL1A1, DNAAF1, EML4, EMSY, EPCAM, EZR, GABRA6, GID4, KEL, KIAA1549, KIF1B, KIF5B, KLHL6, LTK, MC1R, MERTK, NCF2, NUTM1, RUNDC1, SAMD9, SDC4, SLC34A2, SNCAIP, SPINK1, STRN, TPM3, VTI1A*
