# Supplementary material for: JCGA: the Japanese version of the Cancer Genome Atlas and its contribution to the interpretation of gene alterations detected in clinical cancer genome sequencing
Source: Hum Genome Var. 2021 Sep 30;8:38. doi: 10.1038/s41439-021-00170-w (PMC8481308; doi:10.1038/s41439-021-00170-w)
Supplement: Supplementary file 5 — Supplementary Table S4. Median tumor mutation burden (TMB) in each principal tumor type [file 41439_2021_170_MOESM5_ESM.pdf]

Supplementary Table S4. Median tumor mutation burden (TMB) in each principal tumor type

Tumor type	Oncotree[1] / TCGA[2] code	Median value of TMB
Lung Neuroendocrine Tumor	LNET	6.25
Lung Squamous Cell Carcinoma	LUSC	5.68
Hepatocellular Carcinoma	HCC	3.68
Colon Adenocarcinoma	COAD	3.59
Rectal Adenocarcinoma	READ	3.12
Cutaneous Squamous Cell Carcinoma	CSCC	2.92
Uterine Endometrioid Carcinoma	UEC	2.77
Esophageal Squamous Cell Carcinoma	ESCC	2.65
Stomach Adenocarcinoma	STAD	2.53
Head and Neck Squamous Cell Carcinoma	HNSC	2.48
Glioblastoma Multiforme	GBM	2.34
Cervical Squamous Cell Carcinoma and Endocervical Adenocarcinoma	CESC	2.31
Renal Clear Cell Carcinoma	CCRCC	1.91
Ovarian Epithelial Tumor	OVT	1.75
Lung Adenocarcinoma	LUAD	1.50
Cholangiocarcinoma	CHOL	1.45
Melanoma	MEL	1.35
Lower-Grade Glioma	LGG	1.25
Breast Invasive Ductal Carcinoma	IDC	1.08
Sarcoma	SARC	1.06
Breast Invasive Lobular Carcinoma	ILC	1.03
Pleural Mesothelioma	PLMESO	0.77
Small Intestinal Carcinoma	SBC	0.77
Gastrointestinal Stromal Tumor	GIST	0.68
Meningioma	MNG	0.60
Salivary Carcinoma	SACA	0.59
Osteosarcoma	OS	0.51
Metaplastic Breast Cancer	MBC	0.45
Thymoma	THYM	0.30
Pancreatic Adenocarcinoma	PAAD	0.24

[1] Oncotree: http://oncotree.mskcc.org/#/home

[2] TCGA (The Cancer Genome Atlas): https://gdc.cancer.gov/resources-tcga-users/tcga-code-tables/tcga-study-abbreviations
